# The feedback control of *UPF3* is crucial for RNA surveillance in plants

**DOI:** 10.1093/nar/gkv237

**Published:** 2015-03-27

**Authors:** Evgeniya Degtiar, Adi Fridman, Dror Gottlieb, Karina Vexler, Irina Berezin, Ronit Farhi, Linoy Golani, Orit Shaul

**Affiliations:** The Mina and Everard Goodman Faculty of Life Sciences, Bar-Ilan University, Ramat-Gan 5290002, Israel

## Abstract

Nonsense-mediated-decay (NMD) is a eukaryotic RNA surveillance mechanism that controls the levels of both aberrant and normal transcripts. The regulation of this process is not well understood. The *Arabidopsis* NMD factor UPF3 is regulated by a negative feedback-loop that targets its own transcript for NMD. We investigated the functional significance of this control for the overall regulation of NMD in *Arabidopsis*. For this, we tested the ability of NMD-sensitive and -insensitive forms of *UPF3*, expressed under the control of *UPF3* promoter, to complement NMD functionality in NMD-mutant plants and investigated their impact in wild-type (WT) plants. The sensitivity of *UPF3* transcript to NMD was essential for efficient complementation of NMD in *upf3* mutants. Upregulated *UPF3* expression in WT plants resulted in over-degradation of certain transcripts and inhibited degradation of other transcripts. Our results demonstrate that, in contrast to mammalian cells, a delicate balance of *UPF3* transcript levels by its feedback loop and by restriction of its transcription, are crucial for proper NMD regulation in *Arabidopsis*. Interestingly, the levels of many small-nucleolar-RNAs (snoRNAs) were decreased in *upf1* and *upf3* mutants and increased upon enhanced *UPF3* expression. This suggests that proper snoRNA homeostasis in *Arabidopsis* depends on the integrity of the NMD pathway.

## INTRODUCTION

Nonsense mediated mRNA decay (NMD) is a eukaryotic quality control mechanism that has a great influence on the plant transcriptome (1–3). The NMD process degrades transcripts having pre-mature termination codons (PTCs), thereby preventing the accumulation of truncated, potentially deleterious proteins. Orthologs of mammalian NMD factors, including the UP-Frameshift (UPF) proteins UPF1, UPF2 and UPF3, the Suppressor with Morphological defects on Genitalia (SMG) proteins SMG1 and SMG7, as well as Exon-Junction Complex (EJC) proteins, were also identified in plants (1,4–12). NMD eliminates not only mutated mRNAs but also transcripts derived from pseudogenes, aberrant mRNA-like non-coding RNAs, alternatively-spliced transcript isoforms and normal transcripts having certain elements (see below) (1–5,7–10,12–17).

The NMD mechanism was studied in detail (18,19). When the translating ribosome reaches a termination codon (TC), it binds the eukaryotic release factors eRF1 and eRF3. Interaction of the poly(A)-binding protein (PABP) with both eRF3 and the eukaryotic initiation factor 4G (eIF4G) leads to normal translation termination and ribosome recycling, which prohibits NMD (20). In transcripts having long 3′ untranslated regions (3′ UTRs), PABP interaction with eRF3 is decreased due to the large physical distance, preventing efficient ribosome recycling by eIF4G (20). Consequently, eRF3 is indicated to interact with the NMD factor UPF1, followed by UPF1 phosphorylation by SMG1 and NMD activation (20,21). It was shown that UPF1 can also bind the mRNA before translation and be displaced to the 3' UTR by the translating ribosome, possibly providing another means of sensing 3′ UTR length (22–24 and references therein). NMD-susceptible transcripts have long 3' UTRs either naturally or due to the appearance of a PTC. The presence of introns >50–55 nt downstream of TCs can also facilitate NMD (25,26), because NMD factors may be present at EJCs. The EJC is a complex of proteins that are deposited on the mRNA 20–24 nucleotides upstream of exon–exon junctions. The NMD factor UPF3, which is a nucleo-cytoplasmic shuttling protein, interacts with the EJC before export of the mRNA to the cytoplasm (27,28). UPF3 also interacts with UPF2, which functions as a bridge between UPF3 and UPF1 (29). UPF1 ability to bind the mRNA also before translation (22–24) explains why, although introns >50–55 nt downstream of TCs increase NMD efficiency, they are not essential for it, and NMD can be activated by long 3′ UTRs alone. NMD can also be activated by upstream open reading frames (uORFs) which, if translated, may result in the prevalence of a long 3′ UTR and potentially downstream introns (30). As in other eukaryotes, plant transcripts were shown to be targeted to NMD by PTCs (1,4–5), long (>300–350 nt) 3′ UTRs (13–14,31–32), uORFs (16,17) and introns >50–55 nt downstream of TCs (8,14,31).

Compared to the substantial knowledge about NMD mechanism, the regulation of this process is less well understood. We and others showed that expression of several eukaryotic NMD factors increases upon depletion of other factors necessary for this process (8,12,17,33–35). This indicates that the affected factors are themselves NMD substrates that are regulated by negative feedback loops. We showed that expression of the *UPF3* gene of the model plant *Arabidopsis thaliana* is higher in *upf1* mutant plants than in wild-type (WT) plants (17). This indicates that *UPF3* expression is regulated by a negative feedback loop that restricts its expression in WT *Arabidopsis* plants, in which NMD functions effectively. It was shown that human *UPF3B* (encoding the main active isoform among the two mammalian UPF3 isoforms) is also regulated by a negative feedback loop that restricts its expression under normal conditions (33). However, this restriction is apparently not critical for the overall regulation of mammalian NMD, since 15- to 20-fold elevated expression of *UPF3B* did not affect NMD in human cells (33). Here we studied the functional significance of the feedback loop dominating the expression of the *Arabidopsis UPF3* gene for the overall regulation of plant NMD. We first identified that the *cis*-element that targets *UPF3* transcript to NMD is its long 3′ UTR. We then tested the ability of NMD-sensitive and -insensitive forms of *UPF3*, expressed under the control of the native *UPF3* promoter, to complement NMD functionality in NMD-mutant plants and investigated their impact in WT plants. We found that the maintenance of NMD functionality in *Arabidopsis* requires that *UPF3* expression will be delicately balanced by the sensitivity of *UPF3* transcript to NMD, as well as by proper restriction of *UPF3* transcription. Microarray and model gene analyses indicated that a moderate (1.5- to 2-fold) increase in *UPF3* transcript levels can lead to NMD impairment for certain transcripts, while the endogenous level of *UPF3* expression is apparently rate-limiting for NMD of other transcripts. This suggests that there are differences in the way NMD is regulated or executed in plant and mammalian cells. As mentioned, an intense (15- to 20-fold) elevation in the transcript levels of *UPF3B* did not impair NMD in mammalian cells (33). This elevated expression also did not enhance NMD, indicating the UPF3B is also not rate-limiting for mammalian NMD (33). Interestingly, we also found that a functional NMD pathway is essential for proper homeostasis of small nucleolar RNAs (snoRNAs) in *Arabidopsis*. Our finding that a non-functional NMD pathway results in snoRNA depletion in *Arabidopsis* is in contrast to a recent observation in human cells, in which NMD impairment does not alter snoRNA content (although it affects the expression of genes that host snoRNAs in their introns) (36,37). Our work uncovers the crucial role of fine-balancing *UPF3* expression for RNA surveillance in *Arabidopsis* and highlights differences in NMD regulation and cellular impact between plant and mammalian cells.

## MATERIALS AND METHODS

### Mutant lines, plant transformation and expression analysis

The WT *A. thaliana* (L.) plants were of the Col-0 accession and the mutant plants were homozygous, and in the Col-0 background. The selection of homozygous progenies of the *Arabidopsis* T-DNA insertion mutants (38) SALK_112922 (*upf1-5*) and SALK_025175 (*upf3-1*) was described (17). The plants were transformed using the floral dip technique. Following selection on 20 μg/ml hygromycin B, about 25 independently-transformed T1 plants were obtained for each construct analyzed in this study in each type of transformed plant. The T2 plants used for expression analysis were germinated on MS (Duchefa Biochemie BV) plates containing 20 μg/ml hygromycin B (for elimination of non-transformed progenies) and grown for 2 weeks in a climate-controlled growth room in a photoperiod of 16 h light and 8 h dark.

### Cycloheximide treatment

For cycloheximide (CHX) treatment in seedlings, seeds were surface sterilized and grown in liquid B5 (Duchefa Biochemie BV) medium for 12 days. The seedlings were vacuum-infiltrated during 3 min with B5 medium including 10 μg/ml CHX (or the ethanol solvent as a control) and then grown with the same medium for 4 h before harvesting. For CHX treatment in cell cultures, 7-day-old *Arabidopsis* cell suspensions [(39), in the Erecta background] were treated with B5 medium including 10 μg/ml CHX (or the ethanol solvent as a control) for 2 h before harvesting.

### Generation of constructs

Sequence information about the *UPF3* gene (Supplementary Figure S1) was obtained from The *Arabidopsis* Information Resource (TAIR). Construct +uORF was similar to construct W+I described previously (17) except that the 5′ UTR of *AtMHX* was replaced with that of *UPF3*. For this, the native sequence of the 5′ UTR of *UPF3* (Supplementary Figure S1) was synthesized by GenScript flanked by the restriction sites of BamHI and NcoI at its 5′ and 3′ ends, respectively. The −uORF construct was created in a similar way, except that an ATG to AAG mutation eliminated the uORF. In both the +uORF and −uORF constructs, the coding sequence of the *Escherichia coli* β-glucuronidase (GUS) reporter was derived from the pCAMBIA1301 vector (Cambia, Australia) and included an intron. This intron was located 53 nt downstream the TC of the uORF of *UPF3*. A similar construct, as well as an intronless GUS, were previously successfully used to demonstrate that the uORF of another gene (*AtMHX*) can expose its transcript to NMD (17). To create constructs N-ter, U-ter and U-ter+I, we used plasmid pJD330 (a kind gift of DR Gallie) that included the CaMV 35S promoter fused to the omega (Ω) 5′ UTR (derived from the coat protein of tobacco mosaic virus), the coding sequence of GUS (that did not include an intron) and the terminator of the *Agrobacterium tumefacience* gene encoding nopaline synthase (NOS). This construct was called N-ter. In the two other constructs, the NOS terminator was replaced with the *UPF3* terminator, with or without the last intron of this gene. The terminator of *UPF3* (Supplementary Figure S1) included the 545 nt long 3′ UTR (with or without the 12th intron) followed by the 300 nt located downstream this 3′ UTR in the *Arabidopsis* genome (to ensure proper termination of transcription). The *UPF3* terminator, flanked with appropriate restriction sites, was synthesized by GenScript. To create construct 35S::NR (NR stands for Non-Regulated; see ‘Results’ section), the coding sequence of *UPF3*, including the first intron of this gene, was synthesized by GenScript and used to replace the GUS coding sequence in construct N-ter. To create construct U3::NR (U3 stands for *UPF3* promoter), a fragment including the promoter and 5′ UTR of *UPF3* (with the point mutation that eliminated the uORF) was synthesized by GenScript and used to replace the promoter and 5′ UTR of construct 35S::NR. The *UPF3* promoter included the 321 bp located upstream of the transcription initiation site of *UPF3* (Supplementary Figure S1). These 321 bp comprised all the untranscribed sequence separating *UPF3* from the presumed TATA box of AT1G33970, the gene located upstream *UPF3* in the *Arabidopsis* genome. AT1G33970 is transcribed in reverse orientation relative to *UPF3*. Thus, no sequence downstream its presumed TATA box was utilized in order to prevent transcription at reverse orientation. The first intron of *UPF3* was maintained in the *UPF3* coding sequence utilized in this work. In construct U3::R (R stands for Regulated; see ‘Results’ section), the native 5′ UTR (including the uORF) and terminator of *UPF3* (including the 12th intron), which were synthesized by GenScript as described above, were used to replace the mutated 5′ UTR and NOS terminator of U3::NR. All parts of construct U3::R were fused exactly as in the native *UPF3* gene and the sequence of this construct is essentially as shown in Supplementary Figure S1B. The three constructs U3::R, U3::NR and 35S::NR had an identical *UPF3* coding sequence, which included the first intron of *UPF3*. All these constructs were cloned into a binary vector that included the coding sequence of *E. coli* hygromycin phosphotransferase, which confers hygromycin B resistance, immobilized into *A. tumefacience* strain *EHA105*, and used for transformation of *Arabidopsis* plants.

### Cloning of gene probes

Total RNA was extracted with the Tri-Reagent (Sigma). Preparation of cDNA was carried out using the Verso cDNA Kit (No. AB-1453/A) according to the manufacturer's instructions and was followed with DNase I treatment. The primer sets used for cloning of partial cDNA sequences and for probe preparation for *UPF3, EF1a* and the two model genes AT3G53400 and AT5G45430 are presented in Supplementary Table S1. The probe of *UPF3* was derived from its coding sequence only (and not from its UTRs).

### RNA extraction and northern blot analysis

Total RNA was extracted with the Tri-Reagent (Sigma). RNA samples were denatured with glyoxal (Sigma) and fractionated on 1% agarose gels. Gel preparation and fractionation were carried out with 10 mM NaPi buffer, pH 7.0. The gels were blotted onto a Zeta-Probe GT membrane (Bio-Rad) with 25 mM NaPi buffer, pH 7.0. RNA was fixed by UV. The membranes were stained by 0.02% methylene blue in 0.3 M sodium acetate (pH 5.5) to visualize the ribosomal RNA and then rinsed in H_2_O. Hybridization was carried out using the DIG-labeling system (Roche Diagnostics GmbH) according to the manufacturer's instructions. Quantification of band densities on gels was performed with the ImageJ program (NIH).

### Quantitative GUS analysis

Quantitative measurement of GUS activity was carried out using the fluorometric assay (40). Plant material was ground in liquid nitrogen and extracted in a buffer containing 50 mM NaPO_4_, pH 7.2, 1 mM Na_2_EDTA, 10 mM β-mercaptoethanol and 10% (v/v) Triton X-100. Following centrifugation (5 min, 14 000 *g*, 4°C), the supernatant was collected and the concentration of proteins was determined using the Bradford reagent (Sigma). Samples including equal amounts of protein were suspended in 250 μl extraction buffer including 1 mM (final concentration) of the fluorescent GUS substrate 4-methylumbelliferyl-β-D-glucuronide (MUG) (Duchefa Biochemie BV). GUS activity was assayed on a 96-well fluorescent plate-reader (Fluoroscan II, Lab Systems) with the excitation wavelength set at 350 nm and the emission wavelength at 460 nm. GUS activity (milli units.mg protein^−1^) was calculated from the slope of the line generated from measures taken at three-minute intervals during two hours, with respect to the slope of commercial pure GUS enzyme (Roche Diagnostics GmbH).

### Microarray analysis

U3::NR-transformed WT plants, as well as empty vector (EV)-transformed *upf1* and *upf3* mutants, were grown two weeks in plates including MS medium containing 20 μg/ml hygromycin B and 1% sucrose. Each of the three plant types was grown in two separate 15 mm perti dish plates. Each plate was divided to two halves: one including one of the three indicated plant types and the second including EV-transformed WT (EV-W) plants. Total RNA was extracted with the Tri-Reagent (Sigma). RNA quality was determined using the Plant Total RNA Nano 6000 assay kit (Agilent Technologies) on the Agilent Technologies 2100 Bioanalyzer. To generate the fluorescently labeled cRNA, total RNA was labeled using the Low Input Quick Amp Labeling kit (Agilent Technologies). *Arabidopsis* microarrays having experimentally validated gene-specific oligonucleotide probes, designed to differentiate between different models of the same gene (based on TAIR10), were designed by OakLabs GmbH and produced by Agilent Technologies. The microarrays were hybridized using the Gene Expression hybridization kit (Agilent Technologies) and scanned using the SureScan Microarray Scanner (Agilent Technologies). The data from all arrays were first subjected to background correction and LOESS within-array normalization using Agilent Feature Extraction software version 9.5.1.1 (Agilent Technologies). The remaining analyses were performed in Partek^®^ Genomics Suite software version 6.6 (Partek Inc.). The log expression ratios produced during the normalization step were analyzed. Data from two biological replicates of each of the three plant types (U3::NR-transformed WT plants, and EV-transformed *upf1* or *upf3* mutants) were compared to eight biological replicates of the EV-W plants and the *P*-values were determined by Student's *t-*tests. The normalized data were analyzed to identify genes with significantly up- or downregulated expression in each of the three plant types compared to EV-W plants, with *P* < 0.05 and a cutoff of 1.5- or 1.2-fold change (FC), as indicated in the text. All *P*-values presented are FDR corrected. The heat map was generated using Euclidean distance as a similarity measure by using Partek^®^ Genomics Suite™ software, version 6.6 (©2012 Partek Inc., St Louis, MO, USA). Information about the properties of the genes analyzed in the microarray, including the sequences of their 5′- and 3′ UTRs and the locations of introns, were obtained from TAIR V10.

### Statistical analysis

One-way analysis of variance (ANOVA) tests were done on log-transformed data, followed by Tukey's Honestly Significant Difference (HSD) post-test. Chi-square goodness-of-fit tests were done with the Yates’ continuity correction. The Benjamini and Hochberg (BH) procedure was used to adjust the *P*-values of the Gene Ontology (GO) terms enriched in the microarray analysis of U3::NR plants.

## RESULTS

### The long 3′ UTR of *UPF3* exposes its transcript to NMD

**Figure 1.**
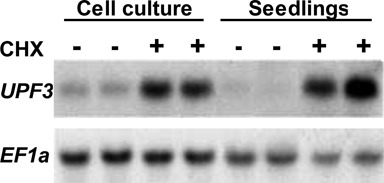
The NMD inhibitor CHX elevates *UPF3* transcript levels. RNA extracted from seedlings and cell cultures of WT *Arabidopsis* plants treated with CHX (+) or with the ethanol solvent (−) was subjected to northern blot analysis using the probes of *UPF3* and the control *EF1a* gene.

We showed that the transcript levels of *UPF3* are higher in mature leaves of *upf1* mutant plants, having depleted *UPF1* levels, than in WT *Arabidopsis* plants (17). A similar observation was made in whole 2-week-old seedlings (Supplementary Figure S2). To further support the conclusion that *UPF3* expression is governed by NMD, we used the translation inhibitor CHX. CHX is often used as an NMD inhibitor, since termination of translation is essential for NMD activation (41). Treatment with CHX increased *UPF3* transcript levels in seedlings and cell cultures of WT *Arabidopsis* plants (Figure 1). The increased levels of *UPF3* transcript in *upf1* mutants and in CHX-treated WT plants, i.e. upon NMD inhibition, supported the conclusion that *UPF3* transcript is controlled by a negative feedback loop that restricts its levels when NMD functions properly.

**Figure 2.**
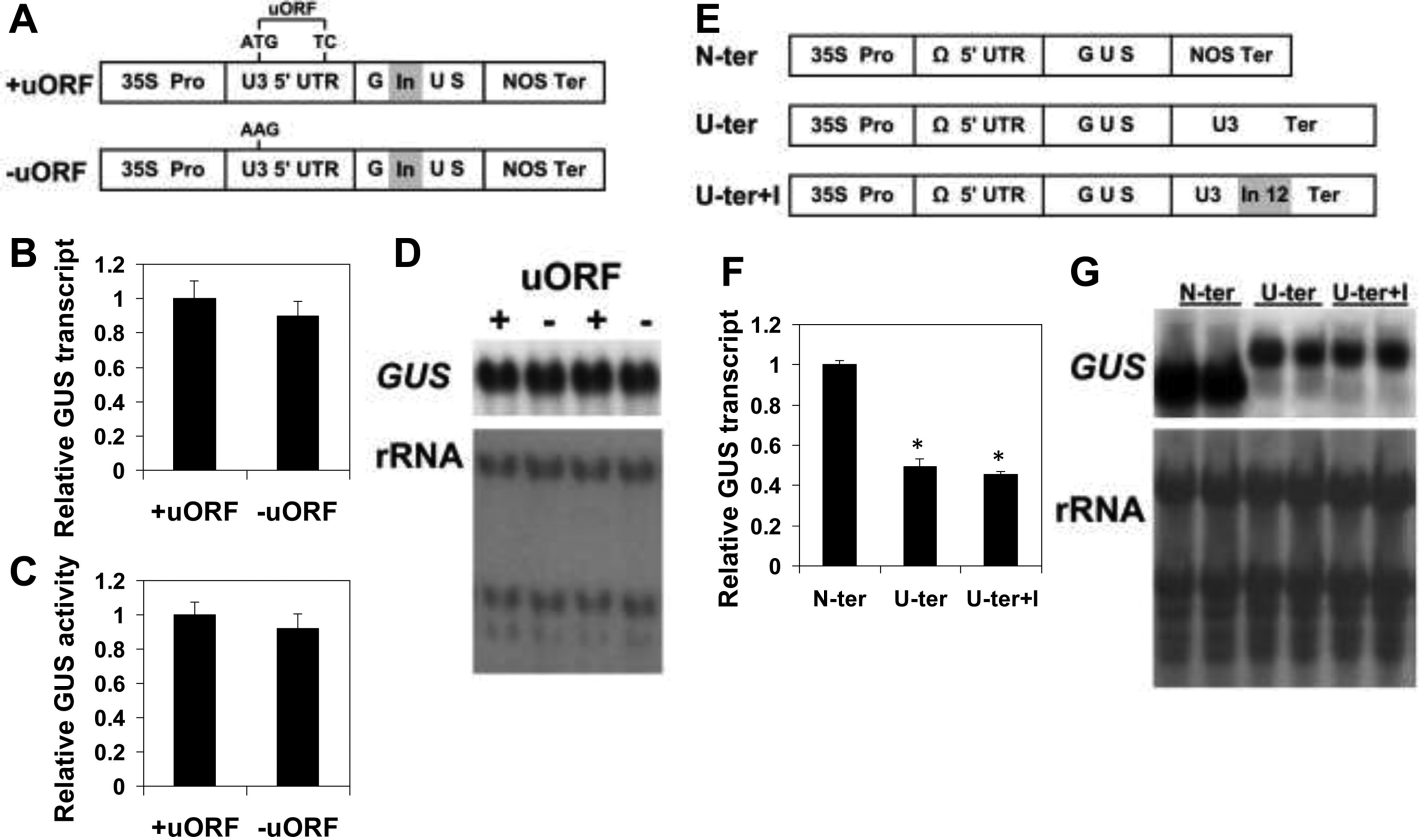
Investigating the impact of the uORF and long 3′ UTR of *UPF3*. (**A**) The constructs used to determine the impact of the uORF. The constructs included the constitutive CaMV 35S promoter (35S Pro), the coding sequence of β-glucuronidase (GUS) that included an intron (indicated by the letters In on gray background; see ‘Materials and Methods’ section), the 5′ UTR of *UPF3* (U3 5′ UTR) and the nopaline synthase terminator (NOS Ter). The +uORF construct had the native 5′ UTR of *UPF3*, while the −uORF construct had an ATG to AAG mutation that eliminated the uORF. (**B** and **C**) *GUS* mRNA (B) and GUS activity (C) of plants expressing the +uORF and −uORF constructs (normalized to the +uORF construct). Plant transformation and expression analysis were carried out as described in the ‘Materials and Methods’ section. Each column presents the mean value ±SEM of *GUS* transcript levels or GUS activity of 2-week-old seedlings grown in two experiments in 20 plates (which constituted 20 biological replicates) that included in total ∼1000 plants. These 1000 plants were composed of ∼40 T2 progeny plants from each of the 25 independent T1 transformants that were regenerated for each construct in each plant. (**D**) Two of the 20 biological replicates that were used for determination of *GUS* transcript levels in (B). The corresponding methylene blue staining of the rRNA is shown below the blot (1 μg of total RNA was loaded in each lane; in all other blots shown in this work, 10 μg of total RNA were loaded in each lane). (**E**) The constructs used to determine the impact of the long 3′ UTR and its intron. The N-ter construct had the short NOS terminator. Construct U-ter+I included the native terminator as well as the 12th intron of *UPF3* (indicated by the letters In12 on gray background). Construct U-ter was similar to U-ter+I but lacked the 3′ UTR intron. Ω 5′ UTR—the 5′ UTR of the coat protein of tobacco mosaic virus, called omega. (**F**) GUS transcript levels (normalized to that of the N-ter construct) of WT plants expressing the constructs illustrated in (E). Each column presents the mean value ±SEM of GUS transcript levels of 2-week-old seedlings grown in two experiments in 10 plates (which constituted 10 biological replicates) that included in total ∼500 plants. These 500 plants were composed of ∼20 T2 progeny plants from each of the 25 independent T1 transformants that were regenerated for each construct in each plant. An asterisk indicates a statistically significant difference (*P* < 0.05) between the N-ter and the other constructs, as determined by Student's *t*-test. (**G**) Two of the 10 biological replicates that were used for determination of *GUS* transcript levels in (F). The corresponding methylene blue staining of the rRNA is shown below the blot. The U-ter and U-ter+I transcripts, which have longer 3′ UTRs, appear in the blot as higher bands compared to the N-ter transcript.

To understand the significance of *UPF3* feedback control for the overall regulation of NMD in plants, it was necessary to identify the elements in *UPF3* transcript that expose it to NMD and to study the consequences of eliminating this regulation. As detailed in the Introduction, elements that can expose plant transcripts to NMD include uORFs, long (>300–350 nt) 3′ UTRs and introns >50–55 nt downstream of TCs. A schematic illustration of the *Arabidopsis UPF3* gene is presented in Supplementary Figure S1A. *UPF3* includes a 48 nt long uORF, a long (545 nt) 3′ UTR and its last intron is localized 19 nt downstream its TC. We investigated the ability of all three elements to expose transcripts including the coding sequence of the GUS reporter gene to NMD in stably transformed *Arabidopsis* plants. Plants expressing reporter constructs that included the 5′ UTR of *UPF3* with (+uORF) or without (−uORF) its uORF (Figure 2A) had similar levels of *GUS* mRNA (Figure 2B and D) and GUS activity (Figure 2C). The fact that GUS transcript levels and GUS activity (which is proportional to the amount of the GUS protein) remained unaltered upon uORF elimination indicated that the uORF of UPF3 does not affect the expression of downstream coding sequences at neither the level of transcript stability nor at the level of translation. The upstream AUG (uAUG) codon of *UPF3* has a very weak Kozak context (U residues at the −3, −2 and +1 positions), making it very likely that this uAUG is not recognized by the ribosome. To conclude, *UPF3* is not targeted for NMD by its uORF (and is also not affected by this uORF at the translational level).

In plants, NMD is induced by 3′ UTRs whose lengths exceed 300–350 nt (13–14,31–32). Long 3′ UTRs were identified in the transcripts of most NMD factors regulated by negative feedback loops, including the *Arabidopsis SMG7* gene and most human NMD factors (8,33,35). The addition of the long 3′ UTRs of the mammalian *UPF1* or *SMG7* genes to reporter gene constructs greatly reduced the transcript levels of these reporter constructs, indicating that the above factors are indeed exposed to NMD by their long 3′ UTRs (33,35). Using a similar approach, we transformed plants with reporter constructs including either the short NOS terminator or the native terminator (and long 3′ UTR) of *UPF3*, with or without its last intron located downstream the TC (Figure 2E). The ability of the two latter constructs having the long *UPF3* 3′ UTR to equally decrease *GUS* transcript levels (Figure 2F and G) suggested that the long 3′ UTR of *UPF3*, but not the 3′ UTR intron (which is located only 19 nt downstream the TC) is the main structural feature that exposes *UPF3* transcript to NMD. To further explore the significance of the length of the 3′ UTR of *UPF3*, two internal deletions were made within this 3′ UTR in the context of the U-ter construct (Supplementary Figure S3). An internal deletion that reduced the 3′ UTR length from 545 to 253 nt resulted in a two-fold elevated expression of the reporter gene construct. This supported the idea that *UPF3* transcript is subjected to NMD by its long 3′ UTR.

### Expression of NMD-sensitive and -insensitive forms of *UPF3* in WT and NMD mutant plants

**Figure 3.**
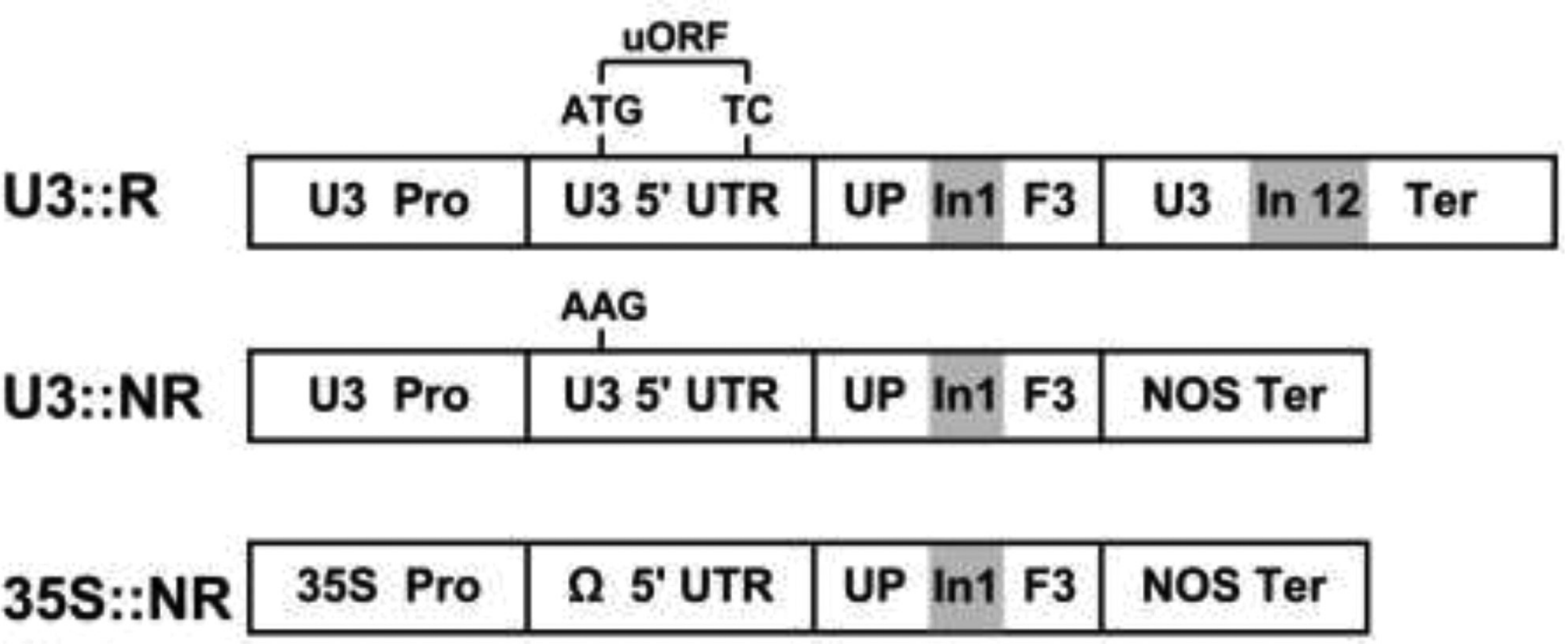
The constructs for expression of *UPF3* in regulated (R) or non-regulated (NR) forms. UPF3—the coding sequence of *UPF3*, which was identical in the three constructs and included the first intron of this gene (indicated by the letters In1 on gray background). See the legend of Figure 2 for the terms of other parts of these constructs.

We aimed to understand the significance of the sensitivity of *UPF3* transcript to NMD for the overall regulation of this surveillance mechanism in *Arabidopsis*. For this, we tested the ability of NMD-sensitive and -insensitive forms of *UPF3* to complement NMD functionality in NMD-mutant plants and investigated their impact in WT plants. To this end, we created three constructs that had an identical *UPF3* coding sequence (which also included the first intron of *UPF3*), but differed in the presence of elements that could expose the transcript to NMD (Figure 3). The first construct included the native promoter, 5′ UTR (including the uORF) and long terminator (including the last intron) of *UPF3* (Figure 3). The native promoter of *UPF3* was used in order to keep the level and sites of expression as similar as possible to those of the endogenous *UPF3* gene. The resulting construct was named U3::R (U3 stands for *UPF3* promoter and R for Regulated form). Notably, this construct was almost identical to the native *UPF3* gene and included all the elements that could target *UPF3* transcript for NMD.

To test the significance of the sensitivity of *UPF3* transcript to NMD, we created a second construct, in which *UPF3* transcript could not be targeted by NMD. This construct, called U3::NR (NR stands for non-regulated form), was similar to U3::R except that all elements that could expose *UPF3* transcript to NMD were eliminated. The long terminator of *UPF3* was replaced with that of NOS, and, to be on the safe side, the uORF was also eliminated by a point mutation (ATG to AAG) (Figure 3). This excluded the possibility that this uORF is targeted for NMD under some unpredicted conditions (however, all our analyses were carried out under the conditions utilized in the analyses presented in Figure 2, in which this uORF was shown not to affect expression).

We also wanted to understand the impact of strong overexpression of *UPF3*. For this, the coding sequence of *UPF3* was expressed under the control of the strong, constitutive CaMV 35S promoter, a 5′ UTR called omega (Ω; derived from the coat protein of tobacco mosaic virus) that was shown to mediate very efficient translation (42) and the NOS terminator. This construct, called 35S::NR, not only lacked elements that could expose *UPF3* transcript to NMD (and was, therefore, also termed NR) but was also expected to lead to strong overexpression of *UPF3*.

Each of the constructs shown in Figure 3, as well as the EV, was stably transformed into plants having mutated *UPF3* or *UPF1* genes and also into WT *Arabidopsis* plants. The *upf3* mutants are homozygous for the *upf3-1* allele and do not have intact *UPF3* mRNA (5). The *upf1* mutants are homozygous for the *upf1-5* allele and have severely reduced, but not completely eliminated expression of *UPF1* (a null mutation in *UPF1* is lethal in *Arabidopsis*) (4,10). About 25 independently-transformed T1 plants were obtained for each construct in each type of transformed plant. The plants used for expression analysis were germinated from mixtures including an equal number of T2 seeds from each of the 25 independent T1 transformants of each construct. In our experience, this approach efficiently compensated for the position effect. The suitability of our approach was validated by the observation that the U3::R construct, which is almost identical to the native *UPF3* gene, efficiently lowered the elevated levels of NMD substrate transcripts in *upf3* mutants (see below) and also restored the mutant phenotype of *upf3* plants into an apparently normal one (supplementary Figure S4).

**Figure 4.**
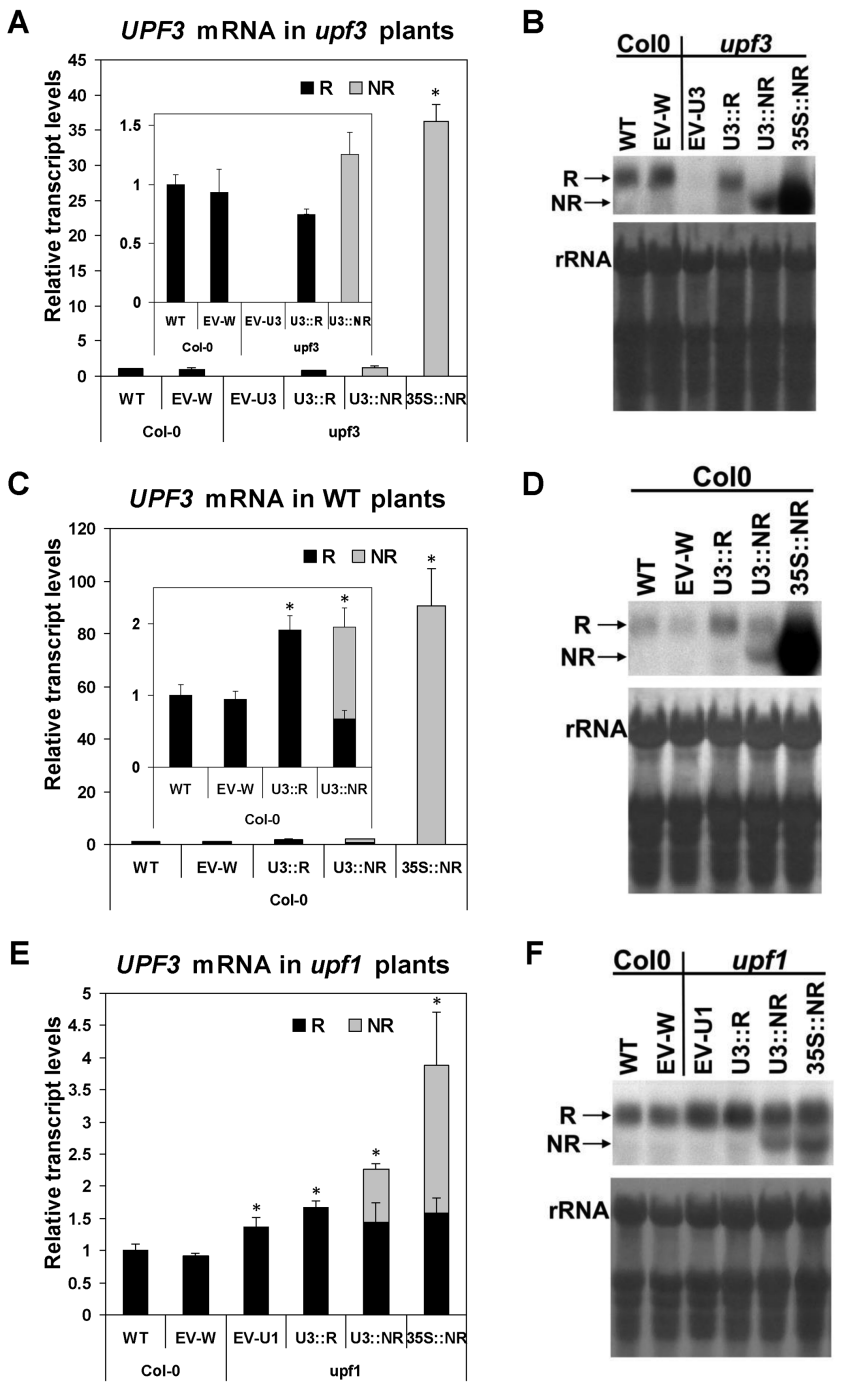
Northern blot analysis of *UPF3* in the transformed plants. (**A**, **C** and **E**) *UPF3* transcript levels (normalized to those of WT plants) of plants expressing the constructs presented in Figure 3 or the empty vector (EV). Each column presents the mean value ±SEM of *UPF3* transcript levels of 2-week-old seedlings grown in 5–7 plates (which constituted 5–7 biological replicates) that included in total ∼300 plants. These 300 plants were composed of ∼12 T2 progeny plants from each of the 25 independent T1 transformants that were regenerated for each construct in each plant. The internal graphs show the constructs with lower expression on a smaller scale. The plant genotypes into which the constructs were transformed (Col-0, *upf3* or *upf1*) are indicated below the graphs. WT—non transformed, wild-type Col-0 plants. The names EV-W, EV-U3 or EV-U1 refer to EV-transformed Col-0, *upf3* or *upf1* plants, respectively. The R and NR forms of *UPF3* transcript are presented by black and gray columns, respectively. When both forms were present in a specific lane, the two forms were also quantified relative to each other and then the NR form was normalized to the R form. All data were also normalized to the intensity of the rRNA staining. The probe of *UPF3* was derived only from its coding sequence (and not from its UTRs). An asterisk indicates a statistically significant difference (*P* < 0.05) in the total levels of *UPF3* transcript between the WT and the indicated plant, as determined by Student's *t*-test. (**B**, **D** and **F**) Each blot presents one of the 5–7 biological replicates that were used for determination of *UPF3* transcript levels in (A, C and E), respectively. The corresponding methylene blue staining of the rRNA is shown below each blot. The plant genotypes into which the constructs were transformed are indicated above the blots.

The levels of *UPF3* transcript in plants expressing each construct were determined by northern blot analysis (Figure 4). *UPF3* transcript levels in *upf3* mutants expressing the U3::R or U3::NR constructs were somewhat lower or higher, respectively, than those of WT plants (or WT plants expressing the EV, named EV-W) (Figure 4A and B). There was a significant (*P* < 0.05 in Student's *t*-test) increase of ∼1.5-fold in *UPF3* transcript levels in *upf3* mutants expressing the U3::NR as compared to the U3::R construct (Figure 4A). The NR form, which has a shorter 3' UTR, appears in the blot as a lower band compared to the R form (Figure 4B). Naturally, no *UPF3* transcript was detected in *upf3* mutants transformed with the EV (EV-U3 plants). As expected, very high levels of the NR form of *UPF3* transcript were observed in *upf3* plants expressing the 35S::NR construct (Figure 4A and B).

The changes in *UPF3* transcript levels in WT plants expressing the three constructs were in line with those seen in the transformed *upf3* mutants, but now the R form was the sum of the endogenous and transformed transcripts (Figure 4C and D). The fact that *UPF3* transcript levels were similar in plants expressing the U3::R and U3::NR constructs may result from NMD impairment in the U3::R plants (see below). The R form could not be quantified in plants expressing the 35S::NR construct due to the high intensity of the NR band.

In *upf1* mutants expressing the EV (EV-U1 plants), *UPF3* transcript levels were higher than those of WT or EV-W plants (Figure 4E and F), as expected based on our previous findings [(17) and Supplementary Figure S2]. Almost no further increase in *UPF3* transcript levels was observed in plants expressing the U3::R construct, possibly due to ability of the *upf1* mutants to still balance *UPF3* expression. Increased *UPF3* transcript levels were seen in U3::NR transformed plants. Expression of the 35S::NR construct was higher than that of the two former constructs, but the extent of this difference was much lower compared to that observed in the transformed *upf3* or WT plants. This was confirmed by direct comparison of *UPF3* transcript levels in transformed WT (Col-0), *upf1* and *upf3* plants expressing the 35S::NR construct (Supplementary Figure S5). Examination of reporter gene constructs that did not include any element of the *UPF3* gene indicated that expression of the 35S promoter is much weaker in *upf1* mutants compared to WT plants or *upf3* mutants (Supplementary Figure S6). As discussed by Moreno *et al*. (43), this finding can be explained by silencing of transgenes including the 35S promoter in the SALK mutant line *upf1-5*. The T-DNA insert in SALK mutants contains a 35S promoter, which resulted in silencing of transgenes having the same promoter in about half of the SALK mutants analyzed (44).

### The sensitivity of *UPF3* transcript to NMD is important for efficient NMD complementation in *upf3* mutants

To determine the impact of the NMD-sensitive and -insensitive forms of *UPF3* on NMD functionality, we selected two model genes: AT3G53400 and AT5G45430 (named hereafter M1 and M2, respectively). There are several indications that these genes are genuine NMD substrates. The transcripts of both M1 and M2 include an element that is highly likely to directly expose them to NMD: an uORF with an evolutionarily conserved peptide (CpuORF). Genes having CpuORFs were found to be hugely over-represented among the common substrates of several NMD mutants in *Arabidopsis* (45). The evolutionally conservation of the CpuORF peptides strongly suggests that these peptides are indeed translated. Hence, their TCs, which are followed by very long 3′ UTRs, can cause NMD (45). The CpuORFs of both M1 and M2 encode rather long peptides (∼40 amino acids) and have strong Kozak-context uAUG codons, which increase the chance that these uAUGs are recognized by the ribosome. Several studies showed that the transcript levels of M1 and M2 are indeed significantly higher in *upf1, upf3* and *smg7* mutants, and in *UPF2*-silenced plants, as compared to WT *Arabidopsis* plants (2,9,45–46). Increased expression of M1 and M2 in NMD mutants was also detected in our experimental conditions (Supplementary Figure S7). Overall, these data suggest that the two model genes are *bona fide* NMD substrates.

Before testing the expression of these model genes in the transformed plants, we examined the transcript levels of the control *EF1a* gene. *EF1a* expression was previously shown to be similar in WT, *upf1* and *upf3* plants (4,17), suggesting that it is not affected by NMD. It was found that the transcript levels of *EF1a* were similar in all the transformed plants (Supplementary Figure S8).

**Figure 5.**
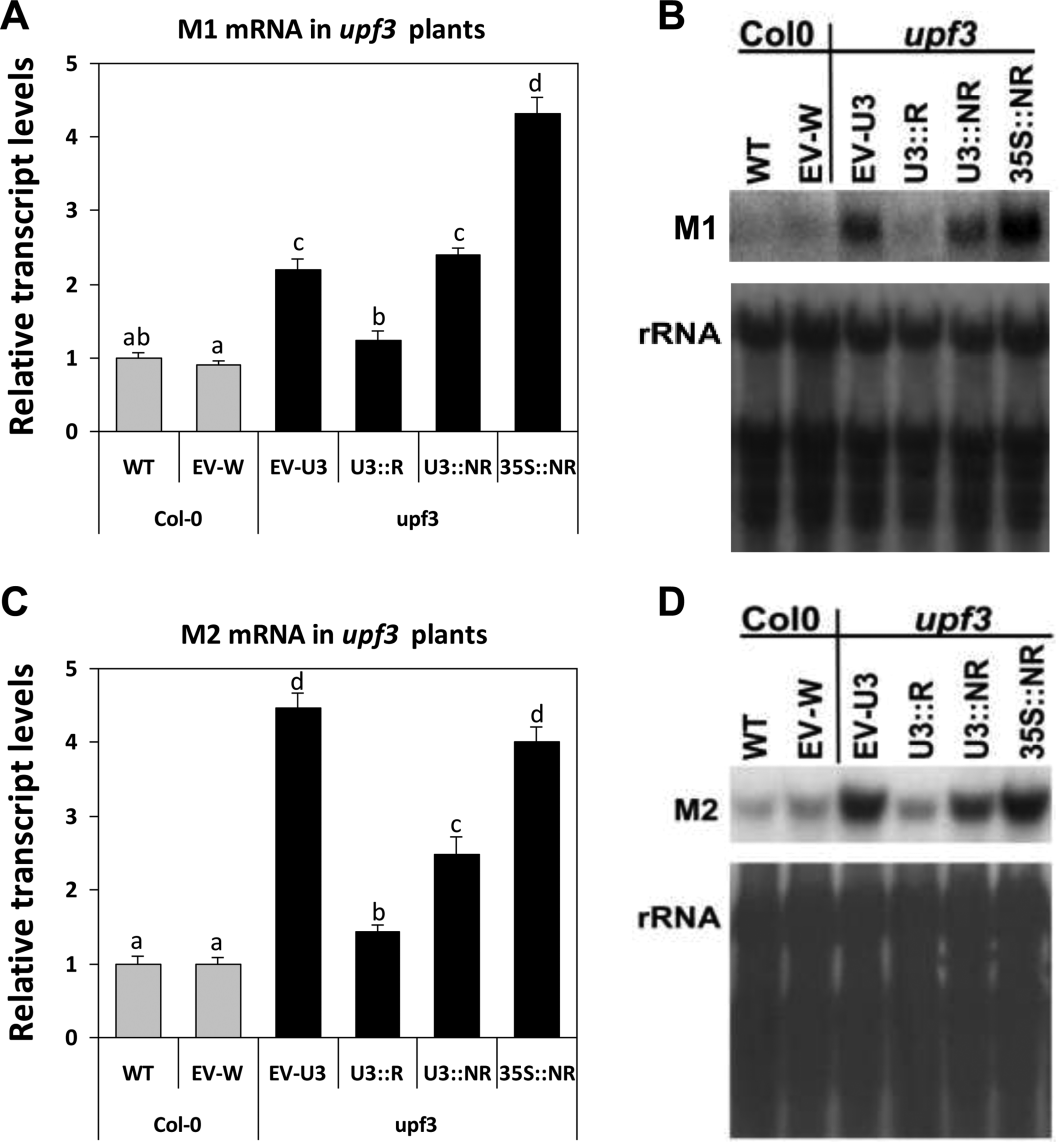
Only the regulated form of *UPF3* can efficiently complement NMD in *upf3* mutants. (**A** and **C**) Following Northern blot analysis of the model genes in the transformed *upf3* and control plants, the transcript levels of M1 (A) and M2 (C) were normalized to those of WT, non-transformed plants. Each column presents the mean value ±SEM of M1 or M2 transcript levels of 2-week-old seedlings that were grown in two independent experiments in 10 plates (which constituted 10 biological replicates) that included in total ∼500 plants. These 500 plants were composed of ∼20 T2 progeny plants from each of the 25 independent T1 transformants that were regenerated for each construct in each plant. The plant genotypes into which the constructs were transformed are indicated below the graphs and by different column colors—gray (Col-0) or black (*upf3*). EV-W and EV-U3 are EV-transformed Col-0 or *upf3* plants, respectively. ANOVA test demonstrated that there were significant differences between the different plants (for M1: *P* < 0.001, *F* = 64.591, df = 5,59; for M2: *P* < 0.001, *F* = 78.178, df = 5,51). Means not sharing a letter on their bars are significantly different at *P* < 0.05 in Tukey's HSD posttest. (**B**) One of the 10 biological replicates that were used for determination of M1 transcript levels in (A). (**D**) One of the 10 biological replicates that were used for determination of M2 transcript levels in (C). The corresponding methylene blue staining of the rRNA is shown below each blot. The plant genotypes into which the constructs were transformed are indicated above the blots.

In *upf3* mutants expressing the U3::R construct, the transcript levels of the two model genes were much lower compared to EV-transformed *upf3* mutants, and similar to, or only slightly higher than those of WT or EV-W plants (Figure 5). This demonstrated the ability of the regulated form of *UPF3* transcript expressed from the U3::R construct to almost completely restore NMD in *upf3* mutants (at least for the two model genes examined).

Interestingly, expression of U3::NR complemented NMD to a much lower extent compared to U3::R (for M2) or not at all (for M1) (Figure 5). As indicated above, *UPF3* transcript levels were only about 1.5-fold higher in *upf3* plants expressing the U3::NR compared to the U3::R construct (Figure 4A). This indicated that even a moderate increase in *UPF3* transcript levels could result in NMD inhibition in *Arabidopsis*. This conclusion was strengthened by the observations in transformed WT plants that had a moderate increase in the regulated (U3::R) form of *UPF3* transcript (see below). The fact that only expression of the NMD-sensitive form of *UPF3* transcript resulted in efficient complementation of NMD in *upf3* mutants indicated that the sensitivity of *UPF3* transcript to NMD is very important for NMD control in *Arabidopsis*.

In *upf3* mutants that expressed the 35S::NR construct, in which *UPF3* transcript levels were particularly high (Figure 4A and B), expression of the NMD target genes examined was either similar to that of *upf3* mutants expressing the EV (for M2) or even higher than that (for M1) (Figure 5). This supports the conclusion that restriction of *UPF3* transcript accumulation is essential for prevention of NMD inhibition. Altogether, the observations in the transformed *upf3* mutants indicated that the NMD feedback loop restricting *UPF3* transcript levels is important for the maintenance of NMD functionality in plants.

### The maintenance of NMD functionality depends not only on post-transcriptional but also on transcriptional control of *UPF3* expression

**Figure 6.**
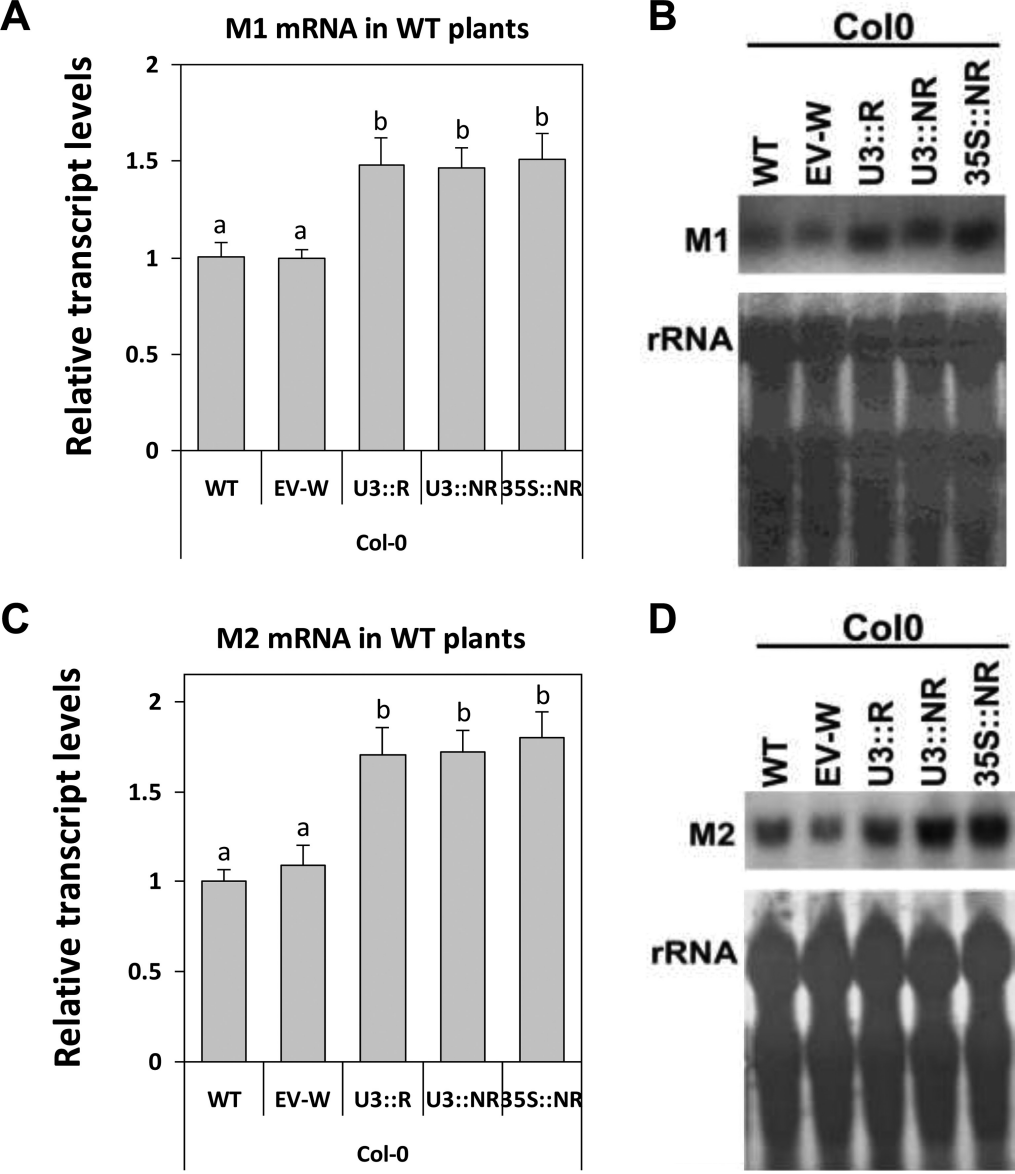
Increased expression of the regulated form inhibits NMD in WT plants. The experimental design is as detailed in the legend of Figure 5. Following northern blot analysis of the model genes in the transformed Col-0 plants, the transcript levels of M1 (**A** and **B**) and M2 (**C** and **D**) were normalized to those of WT, non-transformed plants. EV-W are EV-transformed Col-0 plants. ANOVA test demonstrated that there were significant differences between the different plants (for M1: *P* < 0.001, *F* = 6.978, df = 4,43; for M2: *P* < 0.001, *F* = 10.229, df = 4,50). Means not sharing a letter on their bars are significantly different at *P* < 0.05 in Tukey's HSD posttest.

In WT plants expressing the three constructs, the exogenous *UPF3* transcript accumulated on top of the endogenous one, resulting in elevated levels of *UPF3* transcript in all three types of transformed plants as compared to WT plants (Figure 4C). The transcript levels of the two model genes were higher in all transformed plants compared to WT or EV-transformed plants (Figure 6). Thus, NMD was inhibited not only by the NMD-insensitive form of *UPF3* but also by the moderate increase in *UPF3* transcript derived from the U3::R construct, which included all the native regulatory elements of the *UPF3* gene. As shown above, the U3::R construct efficiently complemented NMD in *upf3* mutants. Yet, increased expression of the same regulated form in WT plants inhibited NMD. This indicates that in general, the maintenance of NMD functionality in *Arabidopsis* requires not only post-transcriptional control of *UPF3* by NMD, but also proper restriction of this gene transcription.

Altogether, the observations in transformed WT and *upf3* plants demonstrate the importance of the mechanisms that restrict the accumulation of *UPF3* transcript for the overall regulation of plant NMD. This is in contrast to the situation in mammalian cells, in which even an intense (15- to 20-fold) increase in the transcript levels of human *UPF3B*, encoding the main active mammalian UPF3 isoform, did not affect NMD (33).

Although the total levels of *UPF3* transcript were much higher in WT plants expressing the 35S::NR construct compared to the two other constructs (Figure 4C and D), the levels of the model genes increased to similar extents upon expression of the three constructs (Figure 6). At least in animals, NMD efficiency was shown to vary in different tissues and cell types (47). It is reasonable to assume that the *UPF3* promoter is preferentially expressed in cells whose NMD process is particularly active. At the same time, since the 35S promoter is constitutive, the high amounts of *UPF3* transcript expressed under the control of the 35S promoter are probably not limited to cells that are exceptionally active in NMD. Thus, a moderate increase in *UPF3* transcript levels in the endogenous sites of this gene expression is apparently more effective in inhibiting NMD than excessive *UPF3* transcript accumulation in other sites. In *upf3* mutants, expression of the two model genes was higher following expression of 35S::NR as compared to the two other constructs (Figure 5). This is presumably because in *upf3* mutants, which lack endogenous *UPF3* expression, the U3::R- and some of the U3::NR-mediated increase in *UPF3* transcript contributed to NMD complementation.

### A delicate balance of *UPF3* expression is also essential under UPF1 limitation

**Figure 7.**
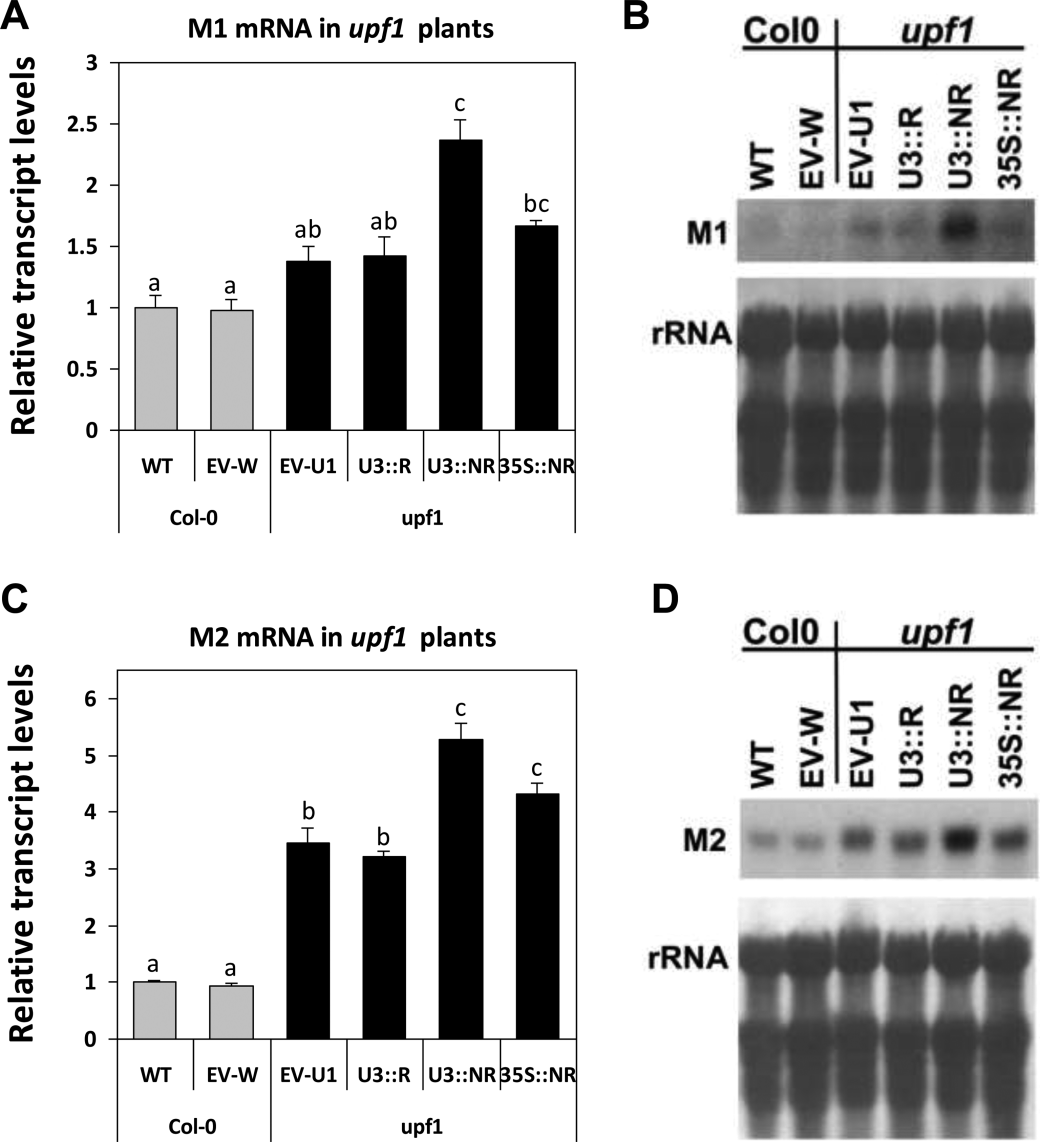
The NMD-sensitivity of *UPF3* is essential under UPF1 limitation. The experimental design is as detailed in the legend of Figure 5. Following northern blot analysis of the model genes in the transformed *upf1* and control plants, the transcript levels of M1 (**A** and **B**) and M2 (**C** and **D**) were normalized to those of WT, non-transformed plants. The plant genotypes into which the constructs were transformed are indicated below the graphs and by different column colors—gray (Col-0) or black (*upf1*). EV-W and EV-U1 are EV-transformed Col-0 or *upf1* plants, respectively. ANOVA test demonstrated that there were significant differences between the different plants (for M1: *P* < 0.001, *F* = 11.838, df = 5,59; for M2: *P* < 0.001, *F* = 225.483, df = 5,54). Means not sharing a letter on their bars are significantly different at *P* < 0.05 in Tukey's HSD posttest.

Crosses with plants having strong or weak alleles for *UPF3* deprivation showed that in homozygous *upf1-5* plants it was not possible to reduce *UPF3* levels to the same extent as that enabled in WT plants [(4); see also ‘Discussion’ section in (17)]. This observation, together with the natural increase in *UPF3* transcript levels observed in *upf1* mutants [(17) and Supplementary Figure S2], supported the assumption that excess expression of *UPF3* can compensate, to a certain extent, for UPF1 limitation (17). It was, therefore, interesting to learn whether further increase in *UPF3* expression could boost NMD complementation under UPF1 limitation. As shown in Figure 4E, almost no increase in *UPF3* transcript levels was seen in *upf1* mutants expressing the U3::R construct. In accord, U3::R expression did not alter the expression of the two model genes compared to *upf1* mutants expressing the EV (EV-U1 plants) (Figure 7). In *upf1* mutants expressing the U3::NR construct, *UPF3* transcript levels were only moderately increased compared to the EV- or U3::R-transformed *upf1* plants (Figure 4E). However, this was accompanied by a significant increase in M1 and M2 transcript levels in the U3::NR transformed *upf1* mutants (Figure 7). Thus, even a small elevation of *UPF3* expression on top of its naturally raised expression in *upf1* mutants could be detrimental to NMD. This suggests that *UPF3* expression must be delicately balanced also under UPF1 limitation and that the sensitivity of *UPF3* transcript to NMD is very important for this balancing [as mentioned, the *Arabidopsis upf1-5* mutants used here still have residual levels of *UPF1* expression (4) and, hence, residual NMD activity].

The total content of *UPF3* transcript was higher in *upf1* plants expressing the 35S::NR as compared to the U3::NR construct (Figure 4E). Nevertheless, the average levels of M1 and M2 transcripts were lower in plants expressing the 35S::NR compared to the U3::NR construct (Figure 7). When the differences in model gene expression were analyzed by Student's *t*-test only between U3::NR- and 35S::NR- expressing *upf1* mutants, they were found to be significant (*P* = 0.001 and 0.01 for M1 and M2, respectively). This supports the previous suggestion that excessive expression of *UPF3* is more effective in inhibiting NMD in the endogenous sites of *UPF3* expression than in other sites. The deleterious impact of the 35S::NR construct was similar to, or higher than that of U3::NR only in the transformed WT or *upf3* plants, in which the extent of overexpression mediated by the 35S promoter was much higher compared to the *upf1* mutants.

### Transcriptome analysis suggests that *UPF3* transcript is delicately balanced between adequate and excessive levels

The observations with the two model genes indicated that the mechanisms restricting *UPF3* transcript accumulation are important for NMD functionality in *Arabidopsis*. To further examine this conclusion, we aimed to determine the impact of de-regulated *UPF3* expression on the whole *Arabidopsis* transcriptome. For this, the transcriptomes of WT plants expressing the U3::NR construct, and of EV-transformed *upf1* and *upf3* mutants, were compared to EV-transformed WT (EV-W) plants using microarray analysis. The analysis was carried out in transformed WT (and not NMD mutant) plants in order to exclude any possibility that the observations for certain NMD substrates might result from inefficient expression of the transformed construct in certain cells (and, hence, from deficient and not increased expression of *UPF3*).

**Figure 8.**
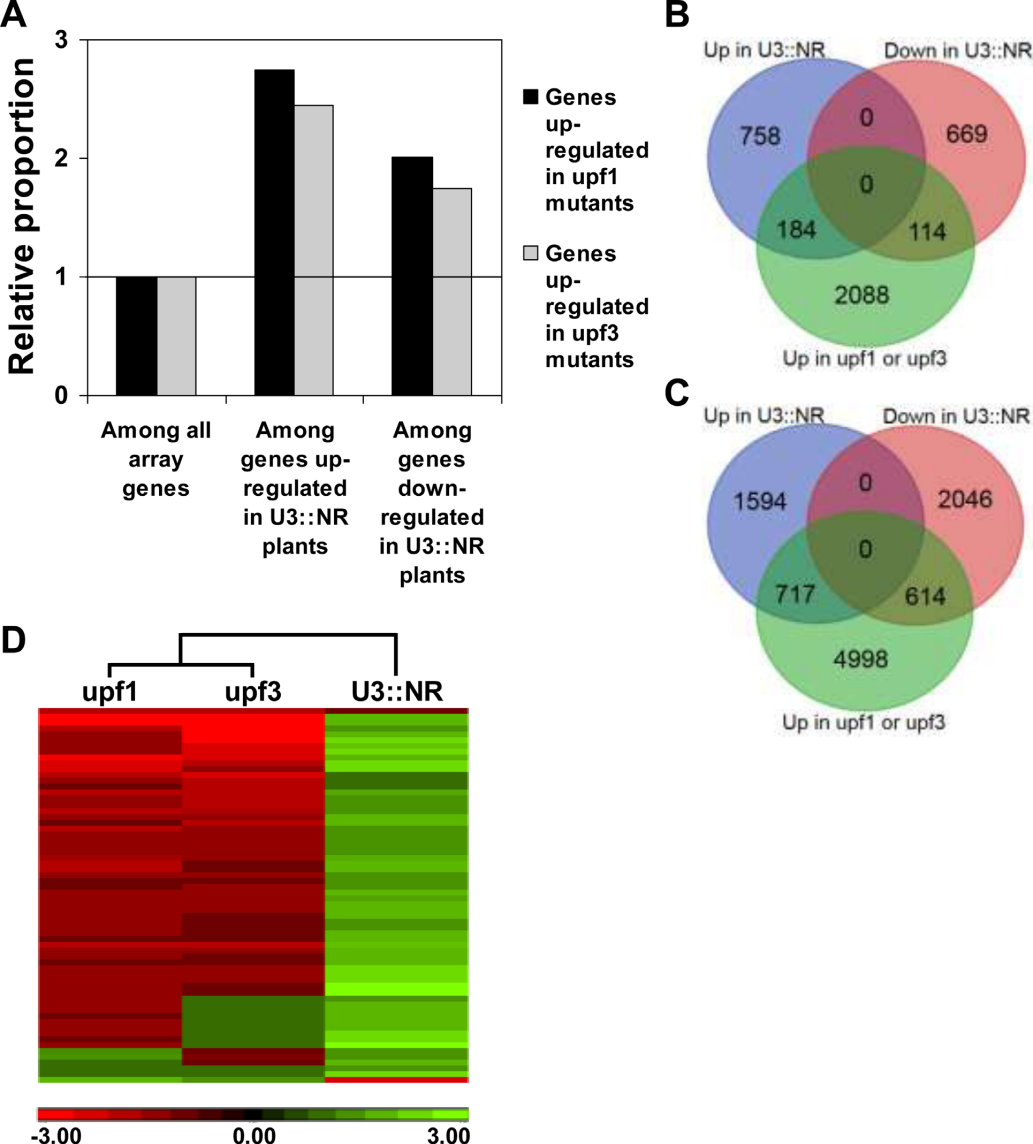
Microarray analysis of U3::NR transformed WT plants. The transcriptomes of U3::NR-transformed WT plants, and of EV-transformed *upf1* and *upf3* mutants (for simplicity, the two latter plant types are indicated in the figure and legend as *upf1* or *upf3* mutants), were compared to that of EV-transformed WT (EV-W) plants. (**A**) The relative proportion of genes upregulated in *upf1* or *upf3* mutants among all array genes (which was given the value of 1) compared to genes up- or downregulated in U3::NR plants. The data presented are of genes whose expression was significantly (*P* < 0.05) altered by at least 1.5-fold. (**B** and **C**) The overlap of genes up- or downregulated in U3::NR plants and genes upregulated in at least one *upf* mutant. The data presented are of genes whose expression was significantly (*P* < 0.05) altered by at least 1.5-fold (B) or 1.2-fold (C). (**D**) Heat map of all 64 snoRNAs in the microarray. The color scale bar below the map presents the fold change (FC). The expression of 59 out of these 64 snoRNAs was differentially regulated in at least one plant type with |FC|>1.4, FDR *P* < 0.1; four snoRNAs were differentially regulated in at least one plant type with |FC|>1.28, FDR *P* < 0.012 and one snoRNA had FC = 1.28, FDR *P* = 0.22 in U3::NR plants (Supplementary Dataset S4).

We first examined whether the group of genes upregulated in U3::NR plants (as compared to EV-W plants) is enriched with genes whose expression is also upregulated in EV-transformed *upf1* or *upf3* mutants grown under the same conditions (as compared to EV-W plants). Figure 8A shows that the group of genes upregulated in U3::NR plants had an about 2.5-fold elevated proportion of genes upregulated in *upf1* or *upf3* mutants, as compared to the proportion of the latter genes among all array genes. This difference was highly significant (*P* = 4.3E-49 in chi-square test; Supplementary Dataset S1). The two model genes showed small but highly significant increased expression in U3::NR plants also in the microarray analysis (Supplementary Dataset S2, model genes). Genes whose expression increases in *upf1* or *upf3* mutants are defined as NMD substrates. The microarray data indicated that, as pre-validated by the northern blot analysis of the M1 and M2 model genes, de-regulated expression of *UPF3* can increase the transcript levels of certain NMD substrates.

Interestingly, an increased proportion of NMD substrates was observed not only among genes upregulated in U3::NR plants, but also among genes downregulated in the latter plants. The group of genes downregulated in U3::NR plants had an about 2-fold elevated proportion of genes upregulated in *upf1* or *upf3* mutants, as compared to the proportion of the latter genes among all array genes. This difference was highly significant (*P* = 5.5E-14 in chi-square test; Supplementary Dataset S1). These data indicated that increased expression of *UPF3* could also decrease the levels of some NMD substrate transcripts, i.e. apparently enhance their NMD.

The analyses presented in Figure 8A were carried out for genes whose expression was altered by an arbitrary cutoff of at least 1.5-fold (with FDR *P* < 0.05) in the indicated plant types. However, Figure 6 indicated that the changes in expression of the model genes M1 and M2 in WT plants expressing the different constructs were apparently small. When the cutoff for analysis of the microarray data was lowered to 1.2-fold (with FDR *P* < 0.05), the results were qualitatively similar to those obtained using the 1.5-fold cutoff, with significant chi-square test values (Supplementary Dataset S2).

The overlap between genes whose expression was altered (either up- or downregulated >1.5-fold) in U3::NR plants and the genes upregulated >1.5-fold in at least one *upf* mutant is schematically illustrated by a Venn diagram (Figure 8B). This overlap was even more apparent using the 1.2-fold cutoff (Figure 8C). The highly significant chi-square tests values detailed above indicated that these overlaps represented highly significant enrichments of the genes altered in U3::NR plants with NMD substrates (i.e. that these overlaps were much larger than those expected to occur between the genes upregulated in *upf* mutants and a random group of array genes).

We then examined the prevalence of potential NMD eliciting features among genes whose expression was altered in the different plants. The features considered were an uORF, a 3′ UTR >300 nt, an intron ≥50 nt downstream the TC or the gene model being a pseudogene. Supplementary Dataset S3 presents this analysis for genes whose expression was altered by at least 1.5-fold (with *P* < 0.05) and for which either the 5′- or 3′ UTR sequence information was available in TAIR (or were pseudogenes). Among these genes, the proportion of those having at least one potential NMD eliciting feature was 57 and 72% for genes upregulated in *upf1* and *upf3* mutants, respectively, and 56 and 59% for genes up- or downregulated in U3::NR plants, respectively. Thus, the proportion of potential NMD features among the genes altered in U3::NR plants was similar to that of *upf1* mutants, although lower than that of *upf3* mutants. The potential NMD features considered did not include transposable elements and potential natural antisense RNAs, for which UTR sequence information was not available, but such transcripts may also be targeted to NMD (2).

The finding that the transcript levels of some NMD substrates were reduced by increased *UPF3* expression could be anticipated based on the known function of UPF3 in NMD activation. Apparently, the endogenous level of *UPF3* expression in WT plants is rate-limiting for NMD of these substrates. The unexpected conclusion that a moderate increase in *UPF3* transcript levels could also inhibit NMD for certain substrates was supported by the combination of the microarray data and the northern blot analysis of the M1 and M2 model genes. The fact that deregulated *UPF3* expression resulted in enhanced NMD of certain substrates and decreased NMD of other substrates suggests that *UPF3* transcript is delicately balanced between adequate and excessive levels (see ‘Discussion’ section).

### A functional NMD pathway is essential for proper homeostasis of *Arabidopsis* snoRNAs

The DAVID functional annotation tool (48) was used to search for GO terms that were over-represented among the transcripts upregulated by at least 1.5-fold (with *P* < 0.05) in U3::NR-transformed WT plants. The highest enrichment score, of 19-fold (with BH adjusted *P*-value of 1E-35), was displayed by a group of 35 genes (Supplementary Dataset S4). Strikingly, all these 35 genes were small nucleolar RNAs (snoRNAs) (Supplementary Dataset S4). We therefore examined the fate of all 64 snoRNAs (all of which having a gene-specific probe) that were represented on the microarray. The expression of most snoRNA genes was increased in U3::NR plants as compared to EV-W plants (Figure 8D and Supplementary Dataset S4). These snoRNAs were apparently not direct NMD substrates, because their levels were not increased in *upf1* or *upf3* mutants grown under the same conditions. On the contrary, there was a strong tendency for decreased levels of these snoRNAs in *upf1* and *upf3* mutants as compared to WT plants (Figure 8D and Supplementary Dataset S4). The reduced expression in *upf1* and *upf3* mutants indicates that a functional NMD pathway is essential for proper homeostasis of *Arabidopsis* snoRNAs.

It was reported that the common genes upregulated in mutants of plant NMD factors have over-representation of proteins involved in the response to pathogens, such as pathogen-related proteins and WRKY transcription factors (45,49). Most of these defense response genes are likely to be indirectly affected by NMD (46). The DAVID analysis of transcripts upregulated in U3::NR plants showed that a group of 43 genes with GO terms related to plant defense was overrepresented by 1.6-fold (with unadjusted *P* = 0.0017) as compared to WT plants (Supplementary Dataset S4). Within these 43 defense genes, analysis of only those for which *either* the 5′- or 3′ UTR sequence was available in TAIR showed that at least one NMD feature could be identified in only 29% of them (compared to 56% among all U3::NR upregulated genes; see above). Thus, both previously characterized NMD mutants (45,49) and U3::NR transformed WT plants have increased proportion of defense genes, most of which apparently being indirect NMD substrates.

## DISCUSSION

The idea that *UPF3* is regulated by a negative feedback loop was based on the increase in *UPF3* transcript levels in *upf1* mutants and in WT plants treated with the NMD inhibitor CHX. It was found that the main structural element that exposes *UPF3* transcript to NMD is its long 3′ UTR. The functional significance of *UPF3* feedback regulation was studied by testing the ability of NMD-sensitive and -insensitive forms of *UPF3* to complement NMD functionality in NMD-mutant plants and investigating their impact in WT plants. The native promoter of *UPF3* was utilized in order to maintain the intensity and spatial control of the expression as similar as possible to those of the endogenous *UPF3* gene. The NMD-sensitive form of *UPF3* almost completely restored NMD in *upf3* mutants for two model genes having features of *bona fide* NMD substrates. However, the NMD-insensitive form of *UPF3* (the U3::NR construct), which mediated only a moderate (about 1.5-fold) increase in *UPF3* transcript levels compared to the U3::R construct, had impaired ability to complement NMD in *upf3* mutants. This demonstrated the significance of the sensitivity of *UPF3* transcript to NMD for the overall regulation of this RNA surveillance mechanism in *Arabidopsis*. The observations in WT plants expressing the U3::R construct showed that NMD impairment occurred even when the transcript levels of the NMD-sensitive form were moderately (2-fold) increased. This indicates that a delicate balance of *UPF3* transcript levels by its feedback loop, as well as by restriction of its transcription, are crucial for proper NMD regulation in *Arabidopsis*.

Transcriptome analysis showed that genes that were either up- or downregulated in U3::NR plants had a significantly higher proportion of genes upregulated in *upf* mutants as compared to the proportion of the latter genes among all array genes (Figure 8A, and chi-square tests in Supplementary Datasets S1 and S2). Genes upregulated in *upf* mutants are defined as NMD substrates. Such substrates can be either direct or indirect targets of NMD. It is difficult to definitely determine based on the presence of specific features which genes are direct targets, because not every gene having a potential NMD features is subjected to NMD and NMD-inducing features are often undefined (50). Yet, the percentages of genes having potential NMD features in genes up- or downregulated in U3::NR plants (56 and 59%, respectively) were similar to that of *upf1* mutants (57%), although lower than that of *upf3* mutants (72%). In addition, although it is difficult to undoubtedly determine for any specific gene whether it is a direct substrate, the fact that the enrichment of genes altered in U3::NR plants with genes upregulated in *upf* mutants is highly significant at a global statistical level (the chi tests), supports the deduction that some of the common genes are direct substrates, while others are similarly affected due to similar changes in direct substrates. As discussed in detail in the ‘Results’ section, the two model genes used in this study are highly likely to be direct NMD substrates.

**Figure 9.**
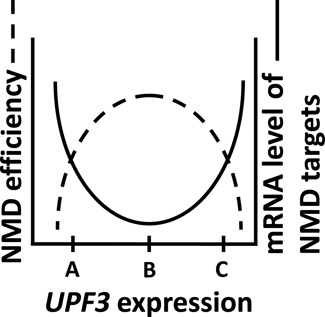
A model for the correlation between *UPF3* expression and NMD efficiency in *Arabidopsis*. In cells or under conditions in which the endogenous level of *UPF3* expression is rate limiting for NMD (point A), an increase in *UPF3* expression can enhance NMD efficiency (presented by the dashed line) and lower the levels of NMD target transcripts (solid line). In cells or under conditions in which *UPF3* expression reaches a certain threshold (point B), further increase in *UPF3* expression (towards point C) can lead to reduced NMD efficiency and, hence, increased levels of NMD target transcripts. The shape of the curves may vary in different cells since the impact of altered *UPF3* expression on NMD efficiency presumably depends also on the expression of other NMD factors.

The fact that a moderate increase in *UPF3* expression reduced the levels of some NMD substrates is in agreement with the known function of UPF3 in NMD enhancement. The unexpected finding that a moderate increase in *UPF3* transcript levels could also inhibit NMD of certain substrates was supported by the combination of the microarray data and the northern blot analysis of the model genes. The distinctive observations for different NMD substrates may result from expression of these substrate genes in different cells, having *a priori* dissimilar levels of expression of the endogenous *UPF3* gene. A model that is coherent with our observations is illustrated in Figure 9. This is a simplified model presenting only *UPF3* impact, but NMD efficiency obviously depends not only on *UPF3* expression but on the interaction between all NMD factors. According to this model, in cells or under conditions in which the endogenous level of *UPF3* expression is still rate limiting for NMD (point A in Figure 9), an increase in *UPF3* expression can enhance NMD efficiency. Restriction of *UPF3* expression at both the transcriptional and post-transcriptional levels is therefore essential for preventing undue, over-degradation of potentially affected transcripts. At the same time, in cells or under conditions in which *UPF3* transcript levels reach a certain threshold (point B in Figure 9), further increase in UPF3 levels can lead to NMD inhibition (point C in Figure 9). To conclude, limited *UPF3* expression (e.g. in *upf3* mutants) inhibits NMD, while a moderate increase in *UPF3* expression can lead to over-degradation of certain transcripts and inhibited degradation of other transcripts. These findings indicate that a delicate balance of *UPF3* transcript levels is crucial for RNA surveillance in *Arabidopsis*.

The downregulation of certain transcripts due to increased expression of *UPF3* suggests that UPF3 is rate limiting for NMD of certain *Arabidopsis* genes. Figure 8A–C show that the degree of overlap with genes upregulated in *upf* mutants was somewhat lower for genes downregulated versus genes upregulated in U3::NR plants. At the same time, the proportion of genes having NMD features was not lower in U3::NR down- versus upregulated genes (59 and 56%, respectively). This raises the possibility that some of the genes downregulated in U3::NR plants are expressed in cells in which *UPF3* expression is normally too low to support efficient NMD (point A in the model presented in Figure 9). Consequently, these genes are not efficiently suppressed in WT plants and are therefore not upregulated in *upf* mutants. The fact that increasing *UPF3* expression is sufficient for reducing the transcript levels of these genes suggests that UPF3 is rate limiting for NMD of certain *Arabidopsis* genes and that modulating *UPF3* expression is apparently sufficient for controlling NMD efficiency for at least certain transcripts.

The findings that the endogenous level of *UPF3* expression is rate-limiting for NMD of certain substrates, whereas a moderate (1.5- to 2-fold) increase in *UPF3* transcript levels can lead to NMD impairment for other substrates suggest that there are differences in the way NMD is regulated or executed in plant and mammalian cells. In human HeLa cells, even a 15- to 20-fold increase in the transcript levels of *UPF3B*, which encodes the main active human UPF3 isoform, did not inhibit NMD (33). This increased expression also did not enhance NMD, indicating that *UPF3B* levels are also not rate-limiting for human NMD (33). Increased expression of non-plant NMD factors was shown to inhibit NMD only in the case of human SMG7, which dephosphorylates and hence deactivates UPF1 (51), or the secondary isoform of human UPF3, called UPF3A, which out-competes the stronger NMD activator UPF3B for participation in NMD complexes (52). An intense elevation in the transcript levels of other human NMD factors either enhanced NMD efficiency (for *SMG1, SMG5* and *SMG6*) or did not alter it (for *UPF1, UPF2* and *UPF3B*) (33).

Interestingly, the group of transcripts that showed the highest enrichment score in U3::NR transformed WT plants was entirely composed of snoRNAs. Most snoRNAs are involved in modifying ribosomal RNAs and spliceosomal small nuclear RNAs (53). The change in snoRNA content apparently did not reflect some pleiotropic impact of extensive *UPF3* overexpression because the extent of increase in *UPF3* transcript levels was moderate, in the range of that naturally occurring in NMD impaired plants (Supplementary Figure S2), i.e. in the range of physiological relevance. Furthermore, the expression of *UPF3* was carried out under the control of its own promoter, i.e. in regions of physiological relevance. The phenotype of the U3::NR transformed WT plants was apparently similar to that of WT plants (data not shown). Moreover, there was a strong tendency for reduced expression of the same snoRNAs in *upf3* and *upf1* mutant plants (Figure 8D). Thus, the correlation between snoRNAs content and the integrity of the NMD pathway was detected not only in the U3::NR plants, which have increased *UPF3* expression, but also in *upf* mutants. The UPF3 protein was previously shown to be enriched in the *Arabidopsis* nucleolus (6). Yet, the changes we observed in snoRNA content are apparently not a simple function of UPF3 levels, because *UPF3* expression is higher compared to WT plants in both U3::NR plants and *upf1* mutants (Supplementary Figure S2). The reduction in snoRNA levels in both *upf3* and *upf1* mutants hints that, while snoRNAs are not direct NMD substrates (otherwise their levels in the mutants should have been increased), snoRNA levels may be collectively affected by putative regulatory or structural protein(s) whose expression is affected by NMD. The expression of these putative protein(s) could be inversely affected in *upf* mutants as compared to U3::NR plants, presumably due to enhanced NMD of some primary target(s) in the latter plants. Specifying such presumed primary target(s) is currently not possible due to the lack of sufficient knowledge about the regulation of snoRNA expression. Nevertheless, our observations in *upf* mutants indicate that a functional NMD pathway is essential for proper homeostasis of *Arabidopsis* snoRNAs.

While a non-functional NMD pathway leads to snoRNA depletion in *Arabidopsis*, it was recently shown that NMD impairment does not alter snoRNA content in human cells. Most vertebrate snoRNA coding units are intronic, nested within introns of protein-coding host genes (53). Interestingly, NMD impairment increases the RNA levels of human snoRNA host genes, but does not affect the levels of their nested snoRNAs (36,37). Regulation of those human host genes by alternative splicing and NMD was suggested to uncouple their expression from that of their nested snoRNAs (37). In contrast to vertebrates, most (except few) *Arabidopsis* snoRNAs are not intronic (53). Many *Arabidopsis* snoRNAs are found in polycistronic clusters (53). Examination of all 64 snoRNA genes on the array showed that the extent of their modification in U3::NR and *upf* mutant plants did not correlate with a specific genomic organization of these genes, i.e. their being intronic or non-intronic or being organized in clusters or as single genes (Supplementary Dataset S4). This suggests that the process that affects snoRNA content upon NMD impairment in *Arabidopsis* does not depend on the genomic organization of the snoRNAs. Further knowledge about the regulation of snoRNA expression will be necessary for identification of the affected process.

Overall, this work demonstrates that the NMD feedback loop dominating *UPF3* transcript levels, as well as fine restriction of *UPF3* transcription, are crucial for NMD homeostasis in plants. As mentioned, an intense elevation in the transcript levels of *UPF3B* did not affect NMD in mammalian cells (33). Further study will be necessary to determine how can an increase in *UPF3* expression inhibit plant NMD and what underlies the contrasting findings in plant and mammalian cells. Yet, some hypotheses can be raised based on the current knowledge about NMD. One possible explanation for our findings is that, when in excess, UPF3 acts in a dominant negative manner, by binding other NMD factor(s) with which it can interact in inappropriate timing or locations, thereby depleting the levels of these factors that can be targeted to other locations or undergo different interactions. It was shown that human UPF3B can interact with UPF2 and with EJC proteins [(54,55); see ‘Introduction’ section]. Similarly, plant UPF3 was shown to bind UPF2 (12). There were some indications that yeast and animal UPF3 proteins could undergo direct interaction with UPF1 (55–57), but plant UPF3 failed to immunoprecipitate with UPF1 in the absence of UPF2 (12). Depletion of plant EJC proteins impaired only intron-based NMD, but did not impair NMD that was dependent on long 3′ UTRs (12). Among the two model genes utilized here, M2 contains many introns, but M1 transcript does not contain any intron. Therefore, it is unlikely that the ability of excess UPF3 to inhibit NMD of the intronless M1 transcript resulted from depletion of EJC proteins. This leaves UPF2 as a possible interacting candidate that could be depleted by excess UPF3. Although the nucleolus is not the site were NMD occurs (13,58), it was shown that the *Arabidopsis* UPF3 is predominantly localized in the nucleolus, UPF2 is found in both the nucleolus and the cytoplasm, and UPF1 is largely cytoplasmic (6). The fact that both UPF3 and UPF2 are enriched in the nucleolus raises the hypothesis that excess UPF3 can inhibit NMD in plants by binding UPF2 in the nucleolus and depleting the amounts of this factor that remain available for shuttling into the cytosol, were NMD occurs. It should also be considered that in contrast to human cells (33), NMD impairment in plants does not lead to a compensatory increase in *UPF2* expression, since our microarray analysis indicated that expression of *UPF2* remained unaltered in *upf1, upf3* or U3::NR plants (data not shown). In addition, the transcript of the *Arabidopsis UPF2* gene (AT2G39260) does not include any potential NMD-inducing feature. The inability of plant cells to elevate *UPF2* expression upon NMD inhibition may enhance the potential of excess UPF3 to deplete UPF2. According to our hypothesis, excess UPF3B does not inhibit NMD in human cells because expression of human *UPF2* can increase upon NMD impairment (33) and/or because human UPF3B and UPF2 are not enriched in the same cellular compartment (27,59). In human cells, UPF3B was predominantly localized in the nucleus (and was not concentrated in the nucleolus), UPF2 was predominantly cytoplasmic or perinuclear, and UPF1 was cytoplasmic (27,59). Further study will be necessary to test this hypothesis.

## SUPPLEMENTARY DATA

Supplementary Data are available at NAR Online.

SUPPLEMENTARY DATA

## References

[1] Yoine M., Ohto M.A., Onai K., Mita S., Nakamura K. (2006). The *lba1* mutation of UPF1 RNA helicase involved in nonsense-mediated mRNA decay causes pleiotropic phenotypic changes and altered sugar signalling in *Arabidopsis*. Plant J..

[2] Kurihara Y., Matsui A., Hanada K., Kawashima M., Ishida J., Morosawa T., Tanaka M., Kaminuma E., Mochizuki Y., Matsushima A. (2009). Genome-wide suppression of aberrant mRNA-like noncoding RNAs by NMD in *Arabidopsis*. Proc. Natl. Acad. Sci. U.S.A..

[3] Drechsel G., Kahles A., Kesarwani A.K., Stauffer E., Behr J., Drewe P., Ratsch G., Wachter A. (2013). Nonsense-mediated decay of alternative precursor mRNA splicing variants is a major determinant of the *Arabidopsis* steady state transcriptome. Plant Cell.

[4] Arciga-Reyes L., Wootton L., Kieffer M., Davies B. (2006). UPF1 is required for nonsense-mediated mRNA decay (NMD) and RNAi in *Arabidopsis*. Plant J..

[5] Hori K., Watanabe Y. (2005). UPF3 suppresses aberrant spliced mRNA in *Arabidopsis*. Plant J..

[6] Kim S.H., Koroleva O.A., Lewandowska D., Pendle A.F., Clark G.P., Simpson C.G., Shaw P.J., Brown J.W.S. (2009). Aberrant mRNA transcripts and the nonsense-mediated decay proteins UPF2 and UPF3 are enriched in the *Arabidopsis* nucleolus. Plant Cell.

[7] Lloyd J.P.B., Davies B. (2013). SMG1 is an ancient nonsense-mediated mRNA decay effector. Plant J..

[8] Nyiko T., Kerenyi F., Szabadkai L., Benkovics A.H., Major P., Sonkoly B., Merai Z., Barta E., Niemiec E., Kufel J. (2013). Plant nonsense-mediated mRNA decay is controlled by different autoregulatory circuits and can be induced by an EJC-like complex. Nucleic Acids Res..

[9] Shi C., Baldwin I.T., Wu J.Q. (2012). *Arabidopsis* plants having defects in nonsense-mediated mRNA decay factors UPF1, UPF2, and UPF3 show photoperiod-dependent phenotypes in development and stress responses. J. Integr. Plant Biol..

[10] Yoine M., Nishii T., Nakamura K. (2006). *Arabidopsis* UPF1 RNA helicase for nonsense-mediated mRNA decay is involved in seed size control and is essential for growth. Plant Cell Physiol..

[11] Riehs N., Akimcheva S., Puizina J., Bulankova P., Idol R.A., Siroky J., Schleiffer A., Schweizer D., Shippen D.E., Riha K. (2008). *Arabidopsis* SMG7 protein is required for exit from meiosis. J. Cell Sci..

[12] Kerenyi Z., Merai Z., Hiripi L., Benkovics A., Gyula P., Lacomme C., Barta E., Nagy F., Silhavy D. (2008). Inter-kingdom conservation of mechanism of nonsense-mediated mRNA decay. EMBO J..

[13] Kalyna M., Simpson C.G., Syed N.H., Lewandowska D., Marquez Y., Kusenda B., Marshall J., Fuller J., Cardle L., McNicol J. (2012). Alternative splicing and nonsense-mediated decay modulate expression of important regulatory genes in *Arabidopsis*. Nucleic Acids Res..

[14] Kertesz S., Kerenyi Z., Merai Z., Bartos I., Palfy T., Barta E., Silhavy D. (2006). Both introns and long 3′-UTRs operate as cis-acting elements to trigger nonsense-mediated decay in plants. Nucleic Acids Res..

[15] Merai Z., Benkovics A.H., Nyiko T., Debreczeny M., Hiripi L., Kerenyi Z., Kondorosi E., Silhavy D. (2013). The late steps of plant nonsense-mediated mRNA decay. Plant J..

[16] Nyiko T., Sonkoly B., Merai Z., Benkovics A.H., Silhavy D. (2009). Plant upstream ORFs can trigger nonsense-mediated mRNA decay in a size-dependent manner. Plant Mol. Biol..

[17] Saul H., Elharrar E., Gaash R., Eliaz D., Valenci M., Akua T., Avramov M., Frankel N., Berezin I., Gottlieb D. (2009). The upstream open reading frame of the *Arabidopsis AtMHX* gene has a strong impact on transcript accumulation through the nonsense-mediated mRNA decay pathway. Plant J..

[18] Kervestin S., Jacobson A. (2012). NMD: a multifaceted response to premature translational termination. Nat. Rev. Mol. Cell Biol..

[19] Popp M.W.-L., Maquat L.E. (2014). The dharma of nonsense-mediated mRNA decay in mammalian cells. Mol. Cells.

[20] Fatscher T., Boehm V., Weiche B., Gehring N.H. (2014). The interaction of cytoplasmic poly(A)-binding protein with eukaryotic initiation factor 4G suppresses nonsense-mediated mRNA decay. RNA.

[21] Kashima I., Yamashita A., Izumi N., Kataoka N., Morishita R., Hoshino S., Ohno M., Dreyfuss G., Ohno S. (2006). Binding of a novel SMG-1-Upf1-eRF1-eRF3 complex (SURF) to the exon junction complex triggers Upf1 phosphorylation and nonsense-mediated mRNA decay. Gene Dev..

[22] Shigeoka T., Kato S., Kawaichi M., Ishida Y. (2012). Evidence that the Upf1-related molecular motor scans the 3'-UTR to ensure mRNA integrity. Nucleic Acids Res..

[23] Zund D., Gruber A.R., Zavolan M., Muhlemann O. (2013). Translation-dependent displacement of UPF1 from coding sequences causes its enrichment in 3' UTRs. Nat. Struct. Mol. Biol..

[24] Kurosaki T., Maquat L.E. (2013). Rules that govern UPF1 binding to mRNA 3' UTRs. Proc. Natl. Acad. Sci. U.S.A..

[25] Zhang J., Sun X.L., Qian Y.M., Laduca J.P., Maquat L.E. (1998). At least one intron is required for the nonsense-mediated decay of triosephosphate isomerase mRNA: a possible link between nuclear splicing and cytoplasmic translation. Mol. Cell. Biol..

[26] Singh G., Rebbapragada I., Lykke-Andersen J. (2008). A competition between stimulators and antagonists of Upf complex recruitment governs human nonsense-mediated mRNA decay. PLoS Biol..

[27] Lykke-Andersen J., Shu M.D., Steitz J.A. (2000). Human Upf proteins target an mRNA for nonsense-mediated decay when bound downstream of a termination codon. Cell.

[28] Kim V.N., Kataoka N., Dreyfuss G. (2001). Role of the nonsense-mediated decay factor hUpf3 in the splicing-dependent exon-exon junction complex. Science.

[29] Singh G., Jakob S., Kleedehn M.G., Lykke-Andersen J. (2007). Communication with the Exon-Junction complex and activation of nonsense-mediated decay by human Upf proteins occur in the cytoplasm. Mol. Cell.

[30] Oliveira C.C., Mccarthy J.E.G. (1995). The relationship between eukaryotic translation and messenger-RNA stability—a short upstream open reading frame strongly inhibits translational initiation and greatly accelerates messenger-RNA degradation in the yeast *Saccharomyces cerevisiae*. J. Biol. Chem..

[31] Hori K., Watanabe Y. (2007). Context analysis of termination codons in mRNA that are recognized by plant NMD. Plant Cell Physiol..

[32] Schwartz A.M., Komarova T.V., Skulachev M.V., Zvereva A.S., Dorokhov Y.L., Atabekov J.G. (2006). Stability of plant mRNAs depends on the length of the 3′-untranslated region. Biochemistry (Mosc).

[33] Huang L.L., Lou C.H., Chan W.K., Shum E.Y., Shao A., Stone E., Karam R., Song H.W., Wilkinson M.F. (2011). RNA homeostasis governed by cell type-specific and branched feedback loops acting on NMD. Mol. Cell.

[34] Rehwinkel A., Letunic I., Raes J., Bork P., Izaurralde E. (2005). Nonsense-mediated mRNA decay factors act in concert to regulate common mRNA targets. RNA.

[35] Yepiskoposyan H., Aeschimann F., Nilsson D., Okoniewski M., Muhlemann O. (2011). Autoregulation of the nonsense-mediated mRNA decay pathway in human cells. RNA.

[36] Weischenfeldt J., Damgaard I., Bryder D., Theilgaard-Moench K., Thoren L.A., Nielsen F.C., Jacobsen S.E., Nerlov C., Porse B.T. (2008). NMD is essential for hematopoietic stem and progenitor cells and for eliminating by-products of programmed DNA rearrangements. Gene Dev..

[37] Lykke-Andersen S., Chen Y., Ardal B.R., Lilje B., Waage J., Sandelin A., Jensen T.H. (2014). Human nonsense-mediated RNA decay initiates widely by endonucleolysis and targets snoRNA host genes. Gene Dev..

[38] Alonso J.M., Stepanova A.N., Leisse T.J., Kim C.J., Chen H., Shinn P., Stevenson D.K., Zimmerman J., Barajas P., Cheuk R. (2003). Genome-wide insertional mutagenesis of *Arabidopsis thaliana*. Science.

[39] May M.J., Leaver C.J. (1993). Oxidative stimulation of glutathione synthesis in *Arabidopsis thaliana* suspension cultures. Plant Physiol..

[40] Breyne P., De Loose M., Dedonder A., Van Montagu M., Depicker A. (1993). Quantitative kinetic analysis of β-glucuronidase activities using a computer-directed microtiter plate reader. Plant Mol. Biol. Rep..

[41] Ishigaki Y., Li X.J., Serin G., Maquat L.E. (2001). Evidence for a pioneer round of mRNA translation: mRNAs subject to nonsense-mediated decay in mammalian cells are bound by CBP80 and CBP20. Cell.

[42] Gallie D.R., Sleat D.E., Watts J.W., Turner P.C., Wilson T.M. (1987). The 5′-leader sequence of tobacco mosaic virus RNA enhances the expression of foreign gene transcripts *in vitro* and *in vivo*. Nucleic Acids Res..

[43] Moreno A.B., de Alba A.E.M., Bardou F., Crespi M.D., Vaucheret H., Maizel A., Mallory A.C. (2013). Cytoplasmic and nuclear quality control and turnover of single-stranded RNA modulate post-transcriptional gene silencing in plants. Nucleic Acids Res..

[44] Daxinger L., Hunter B., Sheik M., Jauvion V., Gasciolli V., Vaucheret H., Matzke M., Furner I. (2008). Unexpected silencing effects from T-DNA tags in *Arabidopsis*. Trends Plant Sci..

[45] Rayson S., Arciga-Reyes L., Wootton L., Zabala M.D., Truman W., Graham N., Grant M., Davies B. (2012). A role for nonsense-mediated mRNA decay in plants: pathogen responses are induced in *Arabidopsis thaliana* NMD mutants. PLoS One.

[46] Rayson S., Ashworth M., Torres Zabala M., Grant M., Davies B. (2012). The salicylic acid dependent and independent effects of NMD in plants. Plant Signal. Behav..

[47] Huang L., Wilkinson M.F. (2012). Regulation of nonsense-mediated mRNA decay. Wiley Indicip. Rev. RNA.

[48] Huang D.W., Sherman B.T., Lempicki R.A. (2009). Systematic and integrative analysis of large gene lists using DAVID bioinformatics resources. Nat. Protoc..

[49] Jeong H.J., Kim Y.J., Kim S.H., Kim Y.H., Lee I.J., Kim Y.K., Shin J.S. (2011). Nonsense-mediated mRNA decay factors, UPF1 and UPF3, contribute to plant defense. Plant Cell Physiol..

[50] Kurosaki T., Li W., Hoque M., Popp M.W., Ermolenko D.N., Tian B., Maquat L.E. (2014). A post-translational regulatory switch on UPF1 controls targeted mRNA degradation. Gene Dev..

[51] Luke B., Azzalin C.M., Hug N., Deplazes A., Peter M., Lingner J. (2007). *Saccharomyces cerevisiae* Ebs1p is a putative ortholog of human Smg7 and promotes nonsense-mediated mRNA decay. Nucleic Acids Res..

[52] Chan W.K., Bhalla A.D., Le Hir H., Nguyen L.S., Huang L.L., Gecz J., Wilkinson M.F. (2009). A UPF3-mediated regulatory switch that maintains RNA surveillance. Nat. Struct. Mol. Biol..

[53] Brown J.W.S., Echeverria M., Qu L.H. (2003). Plant snoRNAs: functional evolution and new modes of gene expression. Trends Plant Sci..

[54] Kadlec J., Izaurralde E., Cusack S. (2004). The structural basis for the interaction between nonsense-mediated mRNA decay factors UPF2 and UPF3. Nat. Struct. Mol. Biol..

[55] Kunz J.B., Neu-Yilik G., Hentze M.W., Kulozik A.E., Gehring N.H. (2006). Functions of hUpf3a and hUpf3b in nonsense-mediated mRNA decay and translation. RNA.

[56] Ivanov P.V., Gehring N.H., Kunz J.B., Hentze M.W., Kulozik A.E. (2008). Interactions between UPF1, eRFs, PABP and the exon junction complex suggest an integrated model for mammalian NMD pathways. EMBO J..

[57] Takahashi S., Araki Y., Ohya Y., Sakuno T., Hoshin S., Kontani K., Nishina H., Katada T. (2008). Upf1 potentially serves as a RING-related E3 ubiquitin ligase via its association with Upf3 in yeast. RNA.

[58] Gohring J., Jacak J., Barta A. (2014). Imaging of endogenous messenger RNA splice variants in living cells reveals nuclear retention of transcripts inaccessible to nonsense-mediated decay in *Arabidopsis*. Plant Cell.

[59] Serin G., Gersappe A., Black J.D., Aronoff R., Maquat L.E. (2001). Identification and characterization of human orthologues to *Saccharomyces cerevisiae* Upf2 protein and Upf3 protein (*Caenorhabditis elegans* SMG-4). Mol. Cell. Biol..

