# Supplementary material for: The feedback control of UPF3 is crucial for RNA surveillance in plants
Source: Nucleic Acids Res. 2015 Mar 27;43(8):4219–35. doi: 10.1093/nar/gkv237 (PMC4417159; doi:10.1093/nar/gkv237)
Supplement: SUPPLEMENTARY DATA [file supp_43_8_4219__index.html]

The feedback control of UPF3 is crucial for RNA surveillance in plants — SUPPLEMENTARY DATA 

# The feedback control of *UPF3* is crucial for RNA surveillance in plants

## SUPPLEMENTARY DATA

**Files in this Data Supplement:**

- Supplementary Data
- Supplementary Dataset S1
- Supplementary Dataset S2
- Supplementary Dataset S3
- Supplementary Dataset S4
