# Supplementary material for: The feedback control of UPF3 is crucial for RNA surveillance in plants
Source: Nucleic Acids Res. 2015 Mar 27;43(8):4219–35. doi: 10.1093/nar/gkv237 (PMC4417159; doi:10.1093/nar/gkv237)
Supplement: SUPPLEMENTARY DATA [file supp_gkv237_Supplementary_Data.pdf]

Supplementary Data

Supplementary Figure S1. *UPF3* gene model and sequence. **(A)** *UPF3* gene model. Introns 2-11, which were not utilized in this work, were not included in the bp count. **(B)** *UPF3* sequences utilized in this work. The sequence used is identical to that of the native *UPF3* gene and flanking sequences (excluding introns 2-11), except five nucleotide alterations (indicated by blue letters) designed to introduce sites for restriction enzymes, without altering the amino acid sequence. The different parts of *UPF3* are indicated by the following colors: black - untranscribed regions, red - 5' and 3' UTR, orange - coding sequence, and purple - the first and 12th introns. The initiation and termination codons of the uORF and main ORF are indicated by bold, underlined letters in red or green, respectively. The positions of the primers used to prepare the *UPF3* gene probe are underlined in the coding sequence. The positions of the AspEI, EcoNI, and StuI restriction sites used for creating the D-370 and D-253 constructs described in Figure S3 are indicated by bold, underlined letters in the 3' UTR.

A

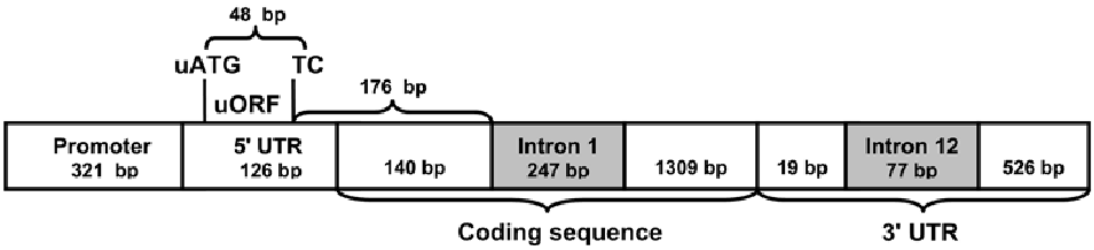

B

cagaatcagtaaaacctgacaagaccagaagtctctgcgaaacaaactcgtactttatcacc
tgcgacgcggatcacctattagattgattatacaccgtacgatgtattattataatcttcat
cctacggtaagcccaataacaagcccatttcacaaactatcagagtaattatctgccgtccg
atgccttaaacttaatccgacggttgaaacatataattaaggcccattagaggcccatata
ggttaaaaagtccacgtagaccgaagattgatttgtctacgggtattttaatttttttctttt
cccagaggaaagggaagagaggaaacaaaaattagggcaaaattagcgtattg**atg**tgtttc
acttttcttcttcttcttcttcatcgtgttcgtgtt**tga**tttgcgactttctccaaggaaaa
tttagggttttcg**ATG**AAGGAACCCCTGCAGAAGAAGAAGGTGGTTGTTCGTCACTTGCCGC
CTTCTCTTTCACAGTCCGATCTCTTATCTCAAATTGATCCTCGTTTCGCTGATCGTTACAAT
TGGGTTTCGTTTCGTCCCGGGAAGTCCAGgttagggttttattttctcgttgggtcttgaat
ttggaattttggtttctatgaattttagggttacgaatttttgtaatttaagaagatgatgg
aaattaaagaagattgtatttgattatgttggaaattgaagattattgtatagatttttgtt
acttagttgattgtagagaaattatacttagtagtattgggttttttttagtgatgtgtgtg
tgtgtttgtgttttttgtggtgtcttcagCTATAAAAATCAGAAGTATTCACGGGCCTATGTA
AGTTTCAAGGCACCAGAAGATGTTTATGAGTTCGCTGCATTTTTCAACGGGCATGTGTTTGT
TAATGAAAAGGGTGCTCAGTTTAAGGCTATAGTTGAATATGCACCTTCTCAGCGTGTTCCGA
AACCGAGTGATAAGAAAGATCCTCGTGAAGGGTCTATTAGTAAAGATCCTGATTATCTTGAG
TTTCTTAAGGTGATTGCACAACCTGTTGAGAATCTTCCTAGTGCTGAAATCCAGTTGGAAAG

AAGAGAAGCTGAGCAGTCTGGTGCTTCAAAAGCGGCTCCCATTGTTACACCTCTTATGGAAT
TCATACGTCAAAAACGTGCCACTGTGATGGGACCCCAGGGTTTATCTGATATTCGAAGAGGA
GGTAGAAGAACCAGAGTAGTCTCTGCAAACAAGCCGAGTCCAAGGCCCTCGAAACGTAACTC
TGAAAAGAAAAAGTATGTGGAAAAAGAAAGTTCAAAGAATGTGCCCCGGAAGACTACAGCAG
ACGTCAGCAGCTCTAAGCCAGATTATCGTCAGTCAAATTCAAGTGGAAAGGAACTACCAGGA
AATGAAACTGCCGCTATCATTGATAGCTCTCCCCCTGGGATAGCATTGACTATGGATTCTGG
GAAGAAAAAGATTTTGCTCCTGAGATCAAAAGACCGAGACAATCCTGATAACCCTCCACCAC
AACCGGAACAGCATATAGACACTAATCTTTCTAGAAACTCCACGGATTCAAGACAAAACCAG
AAGAGTGATGTTGGTGGGAGGTTGATCAAGGGAATACTTCTGAGAAATGACTCTCGACCGAG
CCAGTCTTCCACTTTTGTGCAGTCTGAGCAAAGAGTGGAACCCTCAGAAGCAGAAAACTACA
AACGACCTTCTCGACCAGCCAACACTCGAGCAGGGAAAGATTATCATACTTCTGGTACCATC
AGTGAGAAGCAAGAGAGGCGTACAAGAAACAAGGATAGACCTGATCGTGTTATGTGGGCTCC
TCGTCGTGATGGTAGTGAGGATCAACCACTATCTTCAGCAGGAAACAATGGAGAAGTGAAAG
ACAGGATGTTCTCTCAAAGATCGGGAGAAGTGGTGAACTCCTCTGGTGGTCACACTCTTGAG
AATGGTTCTGCCAGACATTCTAGTCGCCGTGTTGGAGGTCGCAATAGAAAAGAAGAGGTGGT
GATTGGCGAGGGTAAAACCTCCCGGAGAGGAAGTGGTGGTGGTCCCAGTTCACATGAGAAGC
AAATGTGGATCCAAAAACCATCATCCGGTACT**TGA**tatattctcttaacatatggtaagctt
cagtcgcactactaccctctcttacttgaaatttgtcaacacagttttatgattgttgtgac
ctttcaggctagctatatgagccattccactggattttgtccttcaatgggaatctatagat
ctcaatcgaaatggggaagtaacattatcaacatcacttgggttttgga**gacttttggtc**ca AspEI
acgttgtaagtcgtagtattgcacataaaagaggccgttctcaaagttaggaatcagctcaa
gatttatgagaagtgagataagaaaaactcgtttgctactg**ccttttgaagg**ccctagcggt EcoNI
tcacggtacctgcatccccccttttggcttgtgcggtgtcacagacaagtgagtgagaagtg
agaacttactctcgagtctgagtaatgaaaataaagtcaaaagtgtatagggagcgggaagc
acagtgtgtcgggttttattttgggttcctgag**aggcct**tagaacttgattgcacgcttta StuI
accatataagagtttgattactttgaagttaatttcttgctaagtaatattttcttgtggaa
gttagtttatttttatatgattagtctatttgtacttatttttgatttcaccaacataa
aagttatgctgaaagtgatttcacgtttagaaagaccatattcttgcgattaggatt
aaagagggttacataaattaatcaaatggtcaaaaattaggaaatgagaactcactt
gtggaagagaagtcataaggataagagaccaatatatatttccccttggggtcgagt
tttttgggcctggcttgttagttgttacaatcgagtgtgatgcgtcccatttcggcg
gagactttgaatgtgtattattgacctttgattcctttgtccatcattt

Supplementary Figure S2. *UPF3* transcript levels in whole seedlings of two-week-old WT (Col-0) or *upf1* mutant plants. **(A)** *UPF3* transcript levels (normalized to those of WT plants) as determined by northern blot hybridization with the *UPF3* gene probe. Each column presents the mean value ±SEM of *UPF3* transcript levels of two-week-old seedlings grown in 10 plates that included in total ~500 plants. **(B)** A representative blot. rRNA, methylene blue staining of the ribosomal RNA.

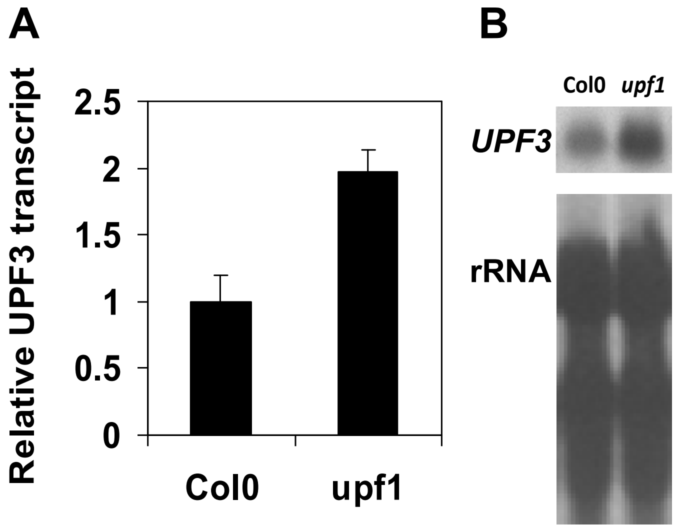

Supplementary Figure S3. GUS expression from reporter genes having internal deletions in the *UPF3* terminator. The basic construct is U-ter illustrated in Figure 2E, which includes the full 545 nt (F-545) 3' UTR of *UPF3*. An internal deletion in the 3' UTR that reduced its length to 370 nt (D-370) did not result in a significant increase in GUS expression, in agreement with data showing that 3' UTR longer than 300-350 nt induce NMD in plants (see Introduction). In contrast, an internal deletion in the 3' UTR that reduced its length to 253 nt (D-253) resulted in a significant (p<0.05 in Student's t-test) increase in GUS expression. GUS expression was normalized to that of the U-ter construct having the full 545 nt 3' UTR (F-545). Each column presents the mean value ±SEM of GUS activity of 20 independently stably transformed three-week-old plants of each construct. Construct D-370 was created by eliminating the region between the EcoNI-StuI restriction sites of the 3' UTR sequence. Construct D-253 was created by eliminating the region between the AspEI-StuI sites of the 3' UTR sequence. These restriction sites are indicated by bold, underlined letters in the 3' UTR sequence presented in Figure S1.

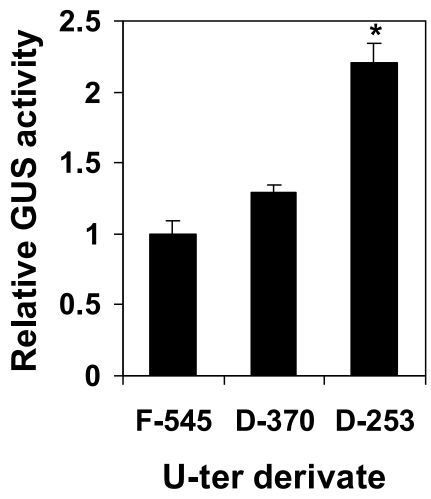

Supplementary Figure S4. The U3::R construct restored the phenotype of *upf3* plants into an apparently WT phenotype. EV-U3 plants (*upf3* mutants transformed with the empty vector) show the narrow leaves and late bolting typical of *upf* mutants, while several independently transformed *upf3* plants expressing the U3::R construct are apparently similar to the EV-W plants (WT plants expressing the empty vector) in terms of leaf phenotype and bolting time.

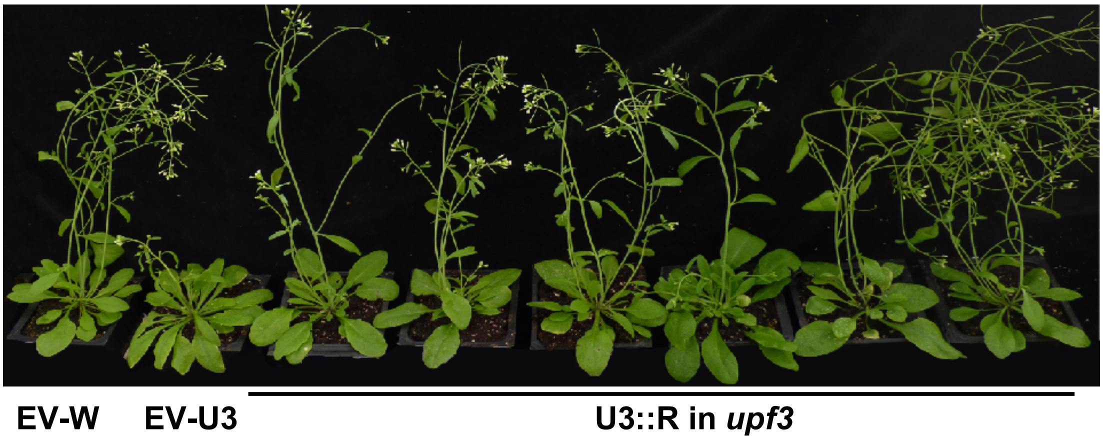

Supplementary Figure S5. Northern blot hybridization of *UPF3* transcript in WT (Col-0), *upf1* and *upf3* plants expressing the 35S::NR construct.

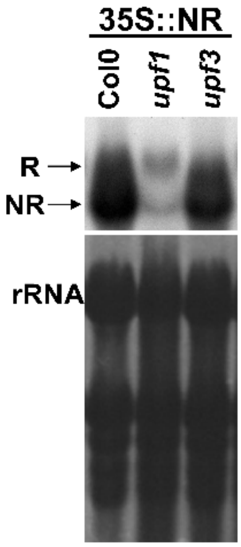

Supplementary Figure S6. Comparison of 35S promoter expression in WT, *upf1*, and *upf3* plants. The N-ter construct illustrated in Figure 2E, which included the 35S promoter but not any element of the *UPF3* gene, was transformed into Col-0, *upf1* and *upf3* plants. The data shown indicate that expression of the 35S promoter is much weaker in *upf1* mutants compared to WT plants or *upf3* mutants. **(A)** GUS transcript levels (normalized to that of WT plants). Plant transformation and expression analysis were carried out as described in the Materials and Methods. Each column presents the mean value ±SEM of *UPF3* transcript levels of two-week-old seedlings grown in 10 plates that included in total ~500 plants. **(B)** A representative northern blot of *GUS* transcript and the corresponding methylene blue staining of the ribosomal RNA (rRNA).

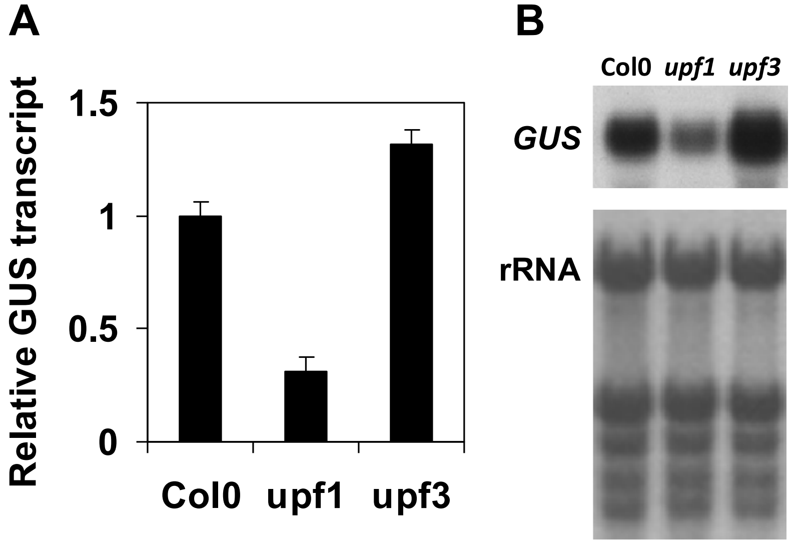

Supplementary Figure S7. Northern blot hybridization of M1 and M2 in non-transformed WT, *upf1*, and *upf3* plants. **(A, C)** The transcript levels of M1 and M2 in non-transformed *upf1* (U1) and *upf3* (U3) mutants, normalized to those of WT plants. Each column presents the mean value ±SEM of two-week-old seedlings grown in 6-10 plates, including 50 plants each. **(B, D)** Representative northern blots of M1 and M2 transcripts.

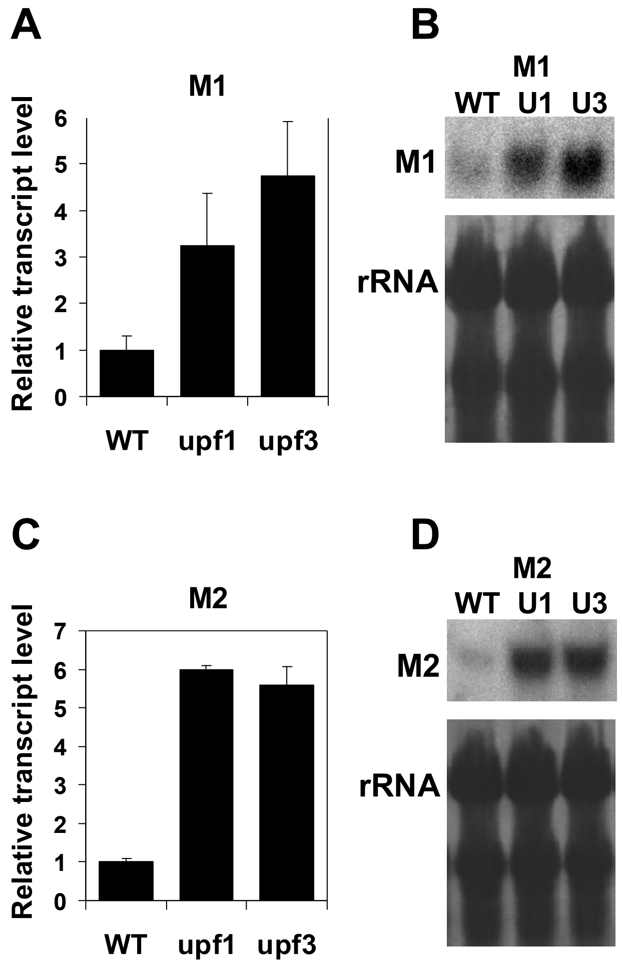

Supplementary Figure S8. Northern blot hybridization of *EF1a* in all transformed plants. The figure shows representative blots (and the corresponding methylene blue staining of the rRNA) of Col-0 **(A)**, *upf1* **(B)** or *upf3* **(C)** plants expressing the constructs shown in Figure 3 or the empty vector (EV). WT - non transformed, wild type Col-0 plants. The names EV-W, EV-U1, or EV-U3 refer to EV-transformed Col-0, *upf1*, or *upf3* plants, respectively.

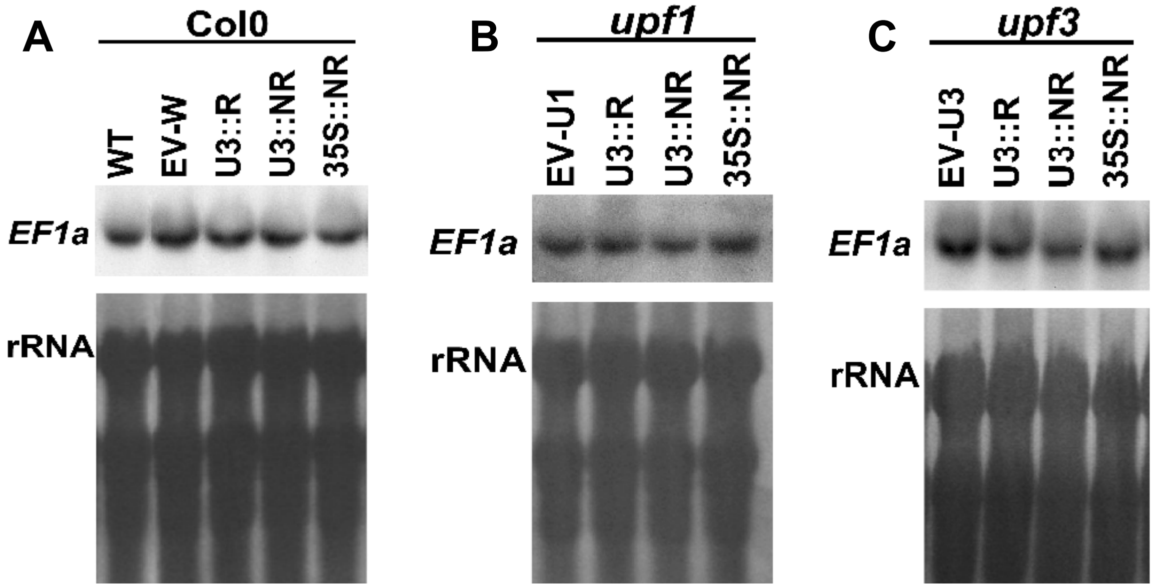

Supplementary Table S1. The primers used for cloning and amplification of probes for northern blot analysis

AGI code	Gene	Orientation	Primer Sequence
AT1G33980	UPF3	Forward	AAGGCACCAGAAGATG
		Reverse	GGATCCACATTTGCTTCTCAT
AT3G53400	M1	Forward	TCCCTGCTTCACTTGTTTAT
		Reverse	AGACGCCACTGTTTCTGAG
AT5G45430	M2	Forward	GCGATGGGTGCTATTATG
		Reverse	GTTGTTGTTGCCTTTGACTT
AT1G07920	EF1a	Forward	CACGTCGATTCTGGAAAGTC
		Reverse	TGATAACACCGACTGCAACAG
